# RNA-sequencing reveals molecular and regional differences in the esophageal mucosa of achalasia patients

**DOI:** 10.1038/s41598-022-25103-7

**Published:** 2022-11-30

**Authors:** Caroline K. Patel, Peter J. Kahrilas, Nathan B. Hodge, Lia E. Tsikretsis, Dustin A. Carlson, John E. Pandolfino, Marie-Pier Tétreault

**Affiliations:** Department of Medicine, Gastroenterology and Hepatology Division, Northwestern University Feinberg School of Medicine, M-336 McGaw Building, 240 East Huron, Chicago, IL 60611-3010 USA

**Keywords:** Transcriptomics, Gastroenterology

## Abstract

Achalasia is an esophageal motility disorder characterized by the functional loss of myenteric plexus ganglion cells in the distal esophagus and lower esophageal sphincter. Histological changes have been reported in the esophageal mucosa of achalasia, suggesting its involvement in disease pathogenesis. Despite recent advances in diagnosis, our understanding of achalasia pathogenesis at the molecular level is very limited and gene expression profiling has not been performed. We performed bulk RNA-sequencing on esophageal mucosa from 14 achalasia and 8 healthy subjects. 65 differentially expressed genes (DEGs) were found in the distal esophageal mucosa of achalasia subjects and 120 DEGs were identified in proximal esophagus. Gene expression analysis identified genes common or exclusive to proximal and distal esophagus, highlighting regional differences in the disease. Enrichment of signaling pathways related to cytokine response and viral defense were observed. Increased infiltration of CD45+ intraepithelial leukocytes were seen in the mucosa of 38 achalasia patients compared to 12 controls. Novel insights into the molecular changes occurring in achalasia were generated in this transcriptomic study. Some gene changes observed in the mucosa of achalasia may be associated with esophagitis. Differences in DEGs between distal and proximal esophagus highlight the importance of better understanding regional differences in achalasia.

## Introduction

Achalasia is a swallowing disorder with an incidence of about 3 in 100,000 individuals per year worldwide^1^. Despite its low incidence, achalasia substantially impacts patients by affecting their work productivity, quality of life and functional status^2^. In advanced cases, achalasia patients may present with dilatation of the esophageal body, which leads to an inability of the esophagus to push food into the stomach, resulting in retained undigested food and liquid. The persistent esophageal distension and food stasis not only lead to symptoms of dysphagia, regurgitation, chest pain, and weight loss, but also result in greater chances of developing squamous cell carcinoma^3,4^.

Recent advances in diagnostic tools for achalasia have provided new insight into the pathogenesis and clinical manifestation of this motility disorder. Functional loss of myenteric plexus ganglion cells in the distal esophagus and LES is believed to be the primary etiology of achalasia, but the cause of the initial reduction of inhibitory neurons remains unknown^5^. Achalasia is categorized into 3 different types based on manometric patterns^6^. Type I is the classic presentation of achalasia and is associated with the complete loss of contractility in the esophageal body. Type II is linked to impaired lower esophageal sphincter (LES) relaxation and pan-esophageal pressurization. Typically, type I achalasia is a later phase of the disease progression compared to type II. Finally, type III might represent a different entity, as it is associated with premature simultaneous spastic distal esophageal contractions. Unfortunately, there is no cure for achalasia. Treatments for achalasia only compensate for the abnormal physiology to better manage the symptoms by reducing LES tone to facilitate esophageal emptying. Despite the advances in diagnostic testing for improved detection of achalasia our knowledge of the molecular changes responsible for achalasia pathogenesis remain poorly understood.

Consistent with our current understanding, most studies on the pathogenesis of achalasia have focused on muscle cells and myenteric plexus neurons. However, there is growing evidence that epithelial cells are sensors of the luminal milieu^7–10^ and can react to long term exposure to food stasis. They are also central players in executing defensive responses. Furthermore, the epithelium is emerging as an important regulator of smooth muscle contraction in many tissues and organs through the release of epithelium-derived factors^11^. Supporting this concept, in-silico simulations using a fully coupled computational model of the esophagus suggest that a highly compliant mucosa is essential for normal esophageal transport function^12^. Some studies have reported histological changes of the esophageal mucosa of achalasia patients, including basal cell hyperplasia and esophagitis, but these studies rely entirely on histological analyses^5,13,14^ and there have been no studies of the human transcriptome in achalasia reported to date. To address this knowledge gap, we investigated whether esophageal mucosa of achalasia patients have distinct gene expression profiles compared to healthy subjects.

## Results

### Subjects

Overall, 22 controls, mean age 32.6 (25–48), 72.7% female, and 37 achalasia patients, mean age 50.3 (25–82), 59.4% female, were included. The controls all had normal endoscopy and 20 had normal motility on HRM. The achalasia cohort entailed 21 with type I achalasia (8/21, 38.0% with dilated esophagus) and 14 with type II achalasia (6/14, 42.9% with dilated esophagus). Table 1 describes subject characteristics by analysis.

**Table 1:** Clinical characteristics of enrolled patients.

	RNA-seq		qPCR		FFPE tissue	
	Healthy (n = 8)	Achalasia (n = 14)	Healthy (n = 4)	Achalasia (n = 23)	Healthy (n = 10)	Achalasia (n = 23)
**Sex, n (%)**						
Male	2 (25.0)	3 (21.4)	4 (100)	12 (52)	4 (40)	12 (52)
Female	6 (75.0)	11 (78.6)	0 (0)	11 (48)	6 (60)	11 (48)
Age, years, mean ± SD	33 ± 8.0	49.9 ± 16.1	29.3 ± 2	50.6 ± 15	33.6 ± 4.4	50.6 ± 15
**Achalasia subtype, n (%)**						
Type I	–	5 (35.7)	–	16 (70)	–	16 (70)
Type II	–	9 (64.3)	–	7 (30)	–	7 (30)
**Dilation status, n (%)**						
Non-dilated	–	7 (50.0)	–	15 (65.2)	–	15 (65.2)
Dilated	–	7 (50.0)	–	8 (34.8)	–	8 (34.8)

### Transcriptomic analyses reveal changes in gene expression levels in the proximal and distal esophagus of achalasia patients

**Figure 1:**
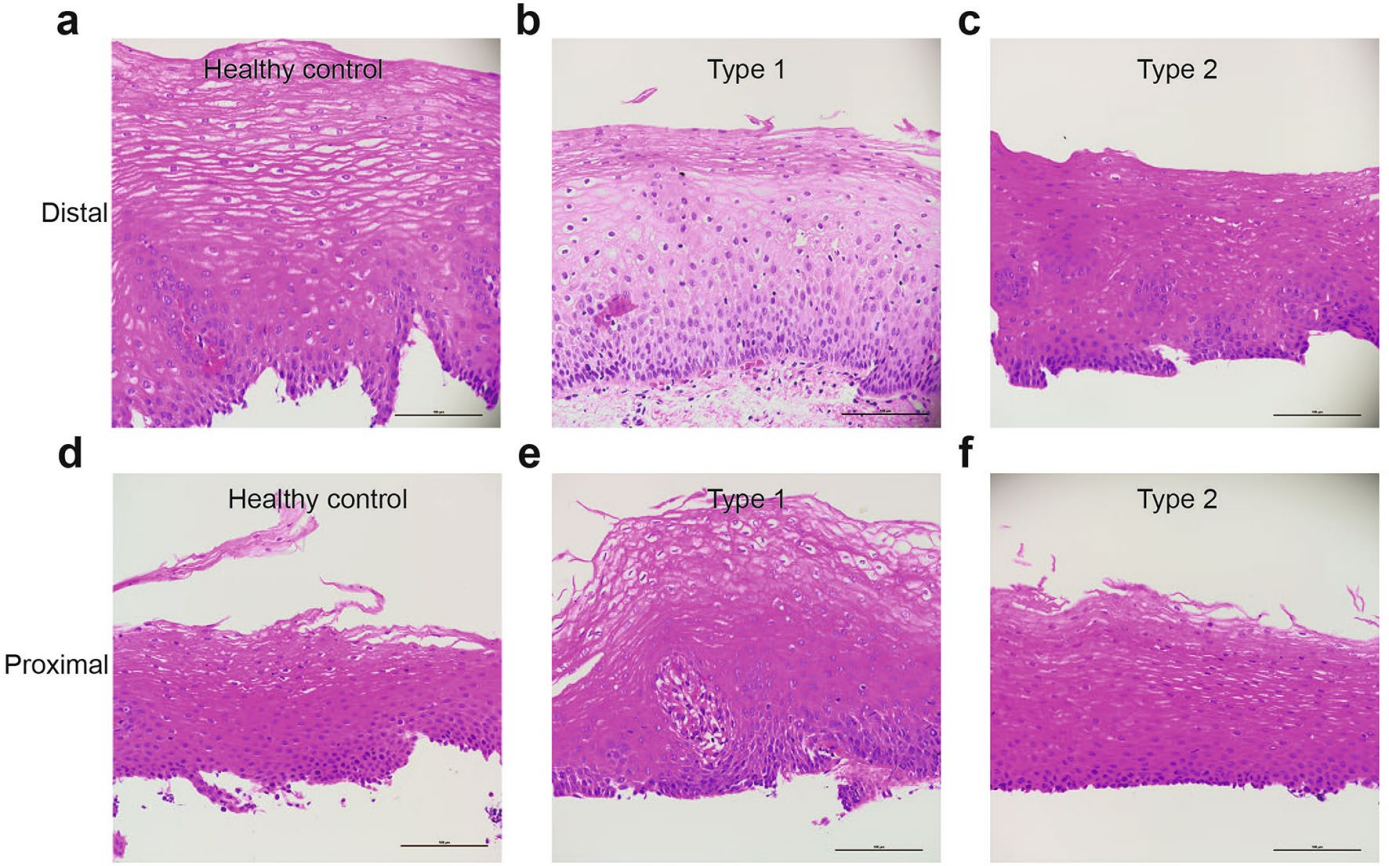
Histology of achalasia and control specimens used for RNA sequencing. H/E staining of esophageal sections from type I (**b**,**e**) and II (**c**,**f**) achalasia show histology changes in distal (**a**–**c**) and proximal (**d**–**f**) esophagus compared to heathy controls (**a**,**d**). Scale bars: 100 μm.

**Figure 2:**
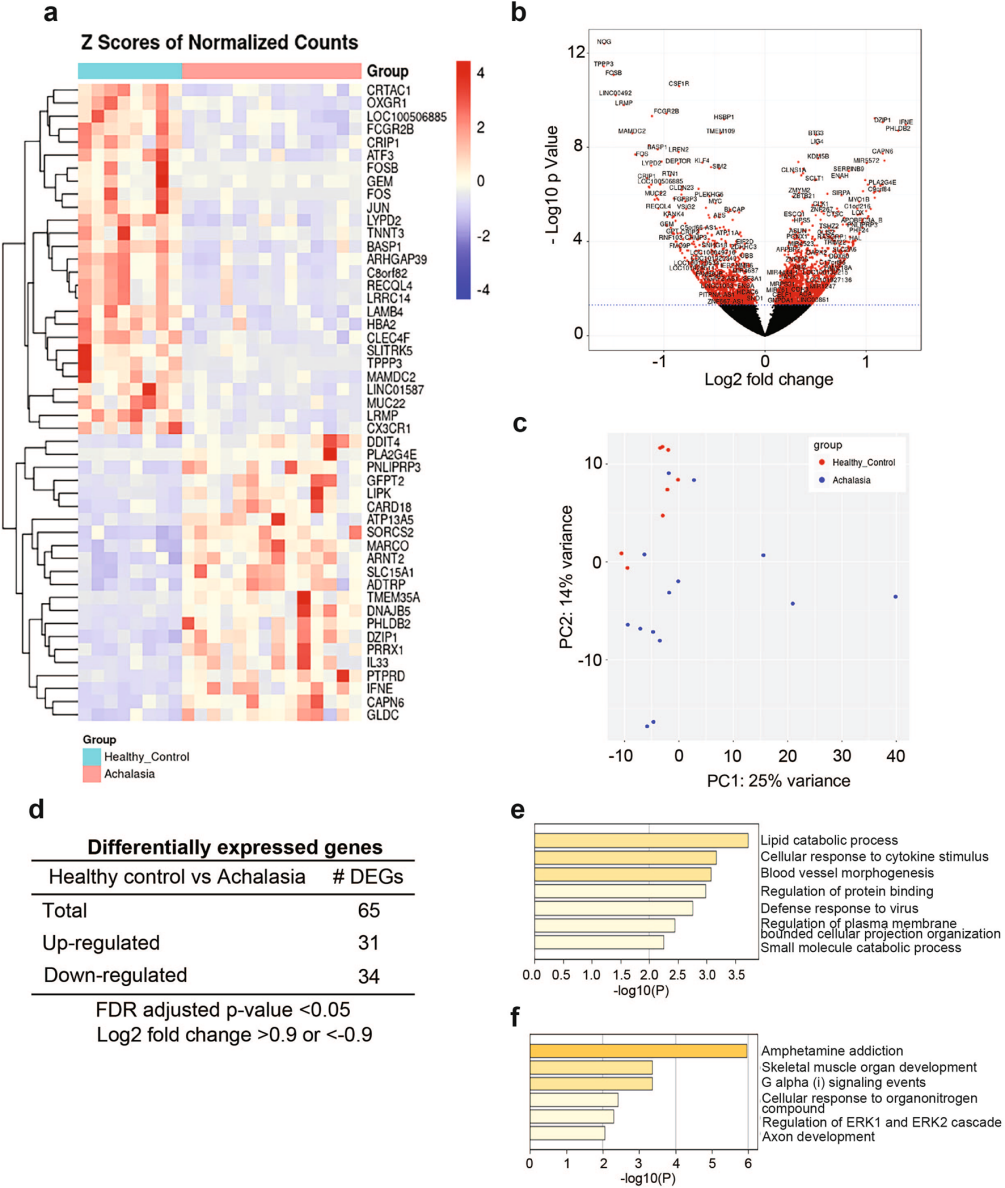
Differential gene expression analysis comparing changes in distal esophageal mucosa of achalasia. (**a**) Heat map of RNA sequencing (RNA-seq) expression data showing differentially expressed genes in achalasia. (**b**) Volcano plot showing the log2 fold change in gene expression in achalasia as well as the statistical significance. (**c**) Principal component analysis of RNA-seq gene expression profiles. red = healthy control, blue = achalasia. (**d**) The number of differentially expressed genes (DEGs) in achalasia based on a log2 fold change of 0.9 and FDR < 0.05. (**e**) A heatmap of the top up-regulated pathways is shown. (**f**) A heatmap of the top down-regulated pathways is shown.

**Figure 3:**
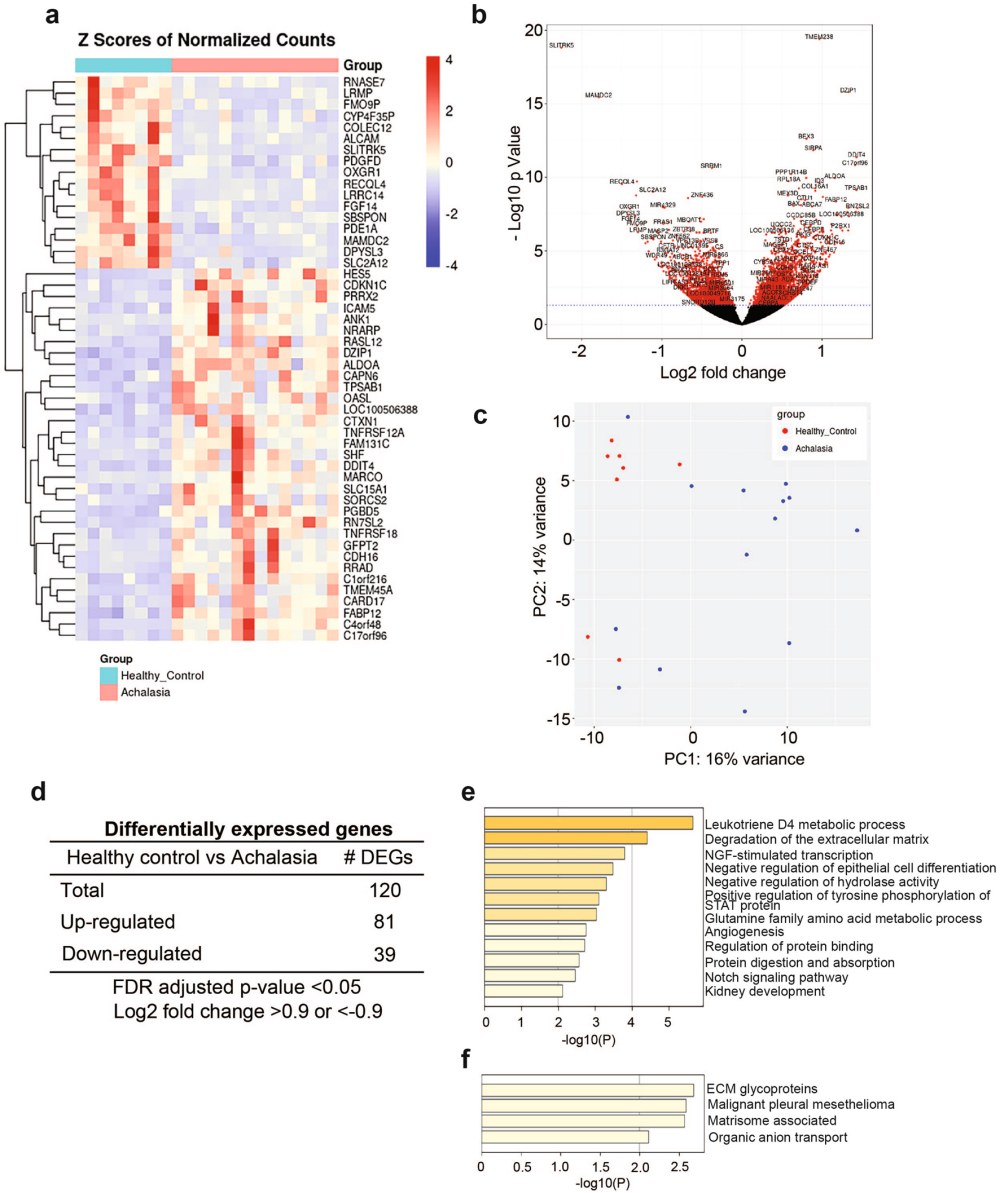
Comparison of differential gene expression analysis in proximal esophageal mucosa of achalasia. (**a**) DEGs in achalasia are shown in a heat map of RNA-seq expression data. (**b**) Volcano plot illustrating the log2 fold change in gene expression in achalasia subjects as well as the statistical significance. (**c**) Principal component analysis was performed using RNA-seq gene expression profiles of achalasia. Red = healthy control, blue = achalasia. (**d**) Differentially expressed genes were selected based on a log2 fold change of 0.9 and FDR < 0.05. (**e**) The heatmap shows the top up-regulated pathways. (**f**) The heatmap shows the top down-regulated pathways.

We performed RNA-seq on esophageal mucosal biopsies from the proximal and distal esophagus of 14 achalasia and 8 healthy controls to identify gene targets that are dysregulated in achalasia (Table 1). Biopsies from the proximal and distal esophagus of these subjects were processed, sequenced, and analyzed separately to determine if regional differences are observed within subjects. Examples of the histology of the biopsies collected and processed are shown in Fig. 1. Differential gene expression was determined compared to healthy controls. Overall, 706 genes were significantly changed in the distal esophagus and 1896 genes were significantly changed in the proximal esophagus (false discovery rate-adjusted p value < 0.05). The gene expression profiles of each sample were visualized and compared using heat maps (Figs. 2a, 3a) and volcano plots (Figs. 2b, 3b). PCA was used to evaluate similarities and differences between controls and achalasia patients, and to determine if each set of subjects can be grouped (Fig. 2c, 3c). As shown in Fig. 2c, PCA plot shows apparent clustering of the achalasia subjects in the distal esophagus, separated from the controls. Data obtained from the proximal esophagus shows some clustering of achalasia patients and controls, despite more variability within each group (Fig. 3c).

### Transcriptomic analysis identifies pathways differentially regulated in achalasia subjects versus healthy controls

After filtering differentially expressed genes (DEGs) between controls and achalasia patients using a threshold of fold change in expression of > 0.9 or < 0.9, we identified 65 DEGs in distal esophagus (31 up-regulated and 34 down-regulated) (Fig. 2d) and 120 DEGs in proximal esophagus (81 up-regulated and 39 down-regulated) (Fig. 3d). Gene Set Enrichment Analysis using Metascape Figs. 2e,f, Figs. 3e,f) and GSEA (Supplementary Figs. 1, 2) were performed using the compilation of genes differentially expressed from achalasia compared to controls. The distal esophageal mucosa of achalasia subjects showed increased gene expression in 7 pathways including those related to cellular response to cytokine stimulus and defense response to virus, when compared to the controls (Fig. 2e). Decreased gene regulation in 6 pathways including those linked to skeletal muscle organ development, G alpha (i) signaling events and regulation of ERK1 and ERK2 cascade and axon development were observed in the distal esophageal mucosa from achalasia patients versus controls (Fig. 2f). On the other hand, mucosa from the proximal esophagus of achalasia had increased gene regulation in 12 pathways including those associated with leukotriene D4 metabolic process, degradation of extracellular matrix, NGF stimulated transcription, negative regulation of epithelial differentiation, positive regulation of tyrosine phosphorylation of STAT protein, angiogenesis, and Notch signaling pathway (Fig. 3e). Finally, down-regulated gene regulation in 4 pathways including ECM glycoproteins and matrisome associated were detected in proximal esophageal mucosa of achalasia patients (Fig. 3f).

### Regional differences in gene expression are observed in the proximal versus distal esophagus of achalasia patients

**Figure 4:**
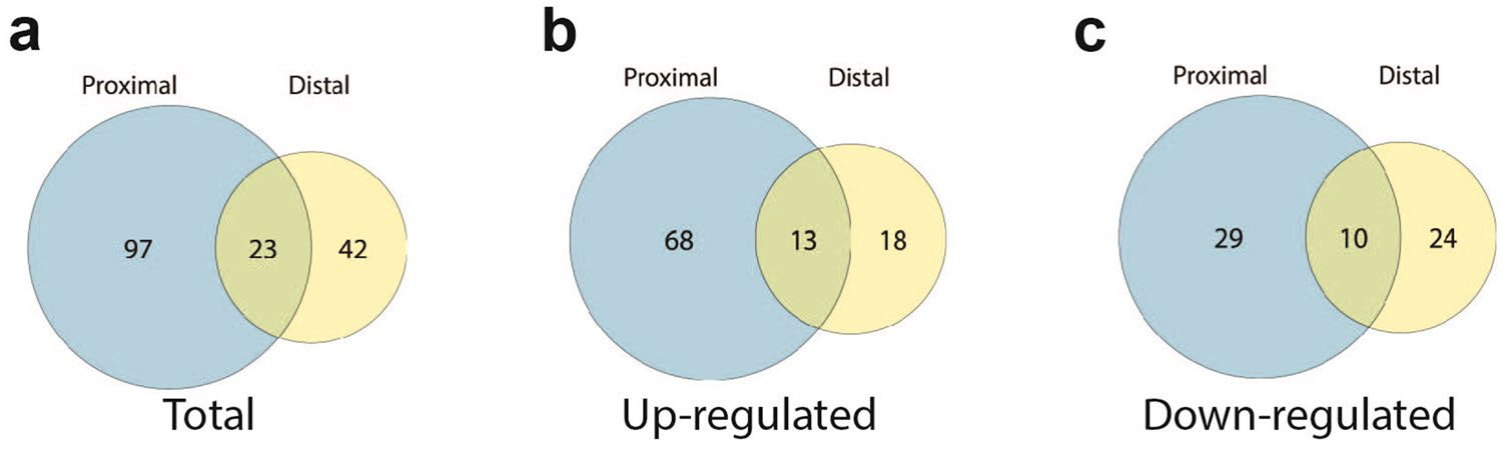
Regional differences in DEGS are observed in proximal and distal achalasia subjects. (**a**) Venn diagram showing overlap of total DEGs in the proximal and distal esophagus of dilated achalasia subjects. (**b**) Venn diagram showing overlap of up-regulated DEGs in the proximal and distal esophagus of achalasia subjects. (**c**) The overlap of down-regulated DEGs between proximal and distal esophagus of achalasia subjects is shown in a Venn diagram.

Our RNA-seq analyses showed differences in the number of DEGs in the distal and proximal esophageal mucosa from achalasia subjects (Figs. 2d and 3d) resulting in differential enrichment of pathways in each region (Figs. 2e,f, 3e,f). As shown in Fig. 4a, only 23 DEGs were found to be common between the distal and proximal esophagus of achalasia patients. 13 of these genes were up-regulated (Fig. 4b) and 10 were down-regulated (Fig. 4c). Examples of common DEGs include *CPA3*, *MAMDC2*, and *CAPN6*. 42 DEGs were exclusive to the distal esophageal mucosa of achalasia patients (Fig. 4a): 18 of these DEGs were up-regulated (Fig. 4b) and 24 were down-regulated (Fig. 4c). The most significantly DEGs only in the distal esophagus include *IL-33*, *IFNε*, *LOX*, and *JUN*. On the other hand, 97 genes were uniquely differentially expressed in the proximal esophageal mucosa of achalasia patients (Fig. 4a): 68 of these genes were up-regulated (Fig. 4b) and 29 were down-regulated (Fig. 4c). This includes change in expression in *CDH16*, *HES5*, *IGFBP3* and *FGF14*.

### Quantitative polymerase chain reaction (qPCR) validates DEGs in achalasia when compared to controls

**Figure 5:**
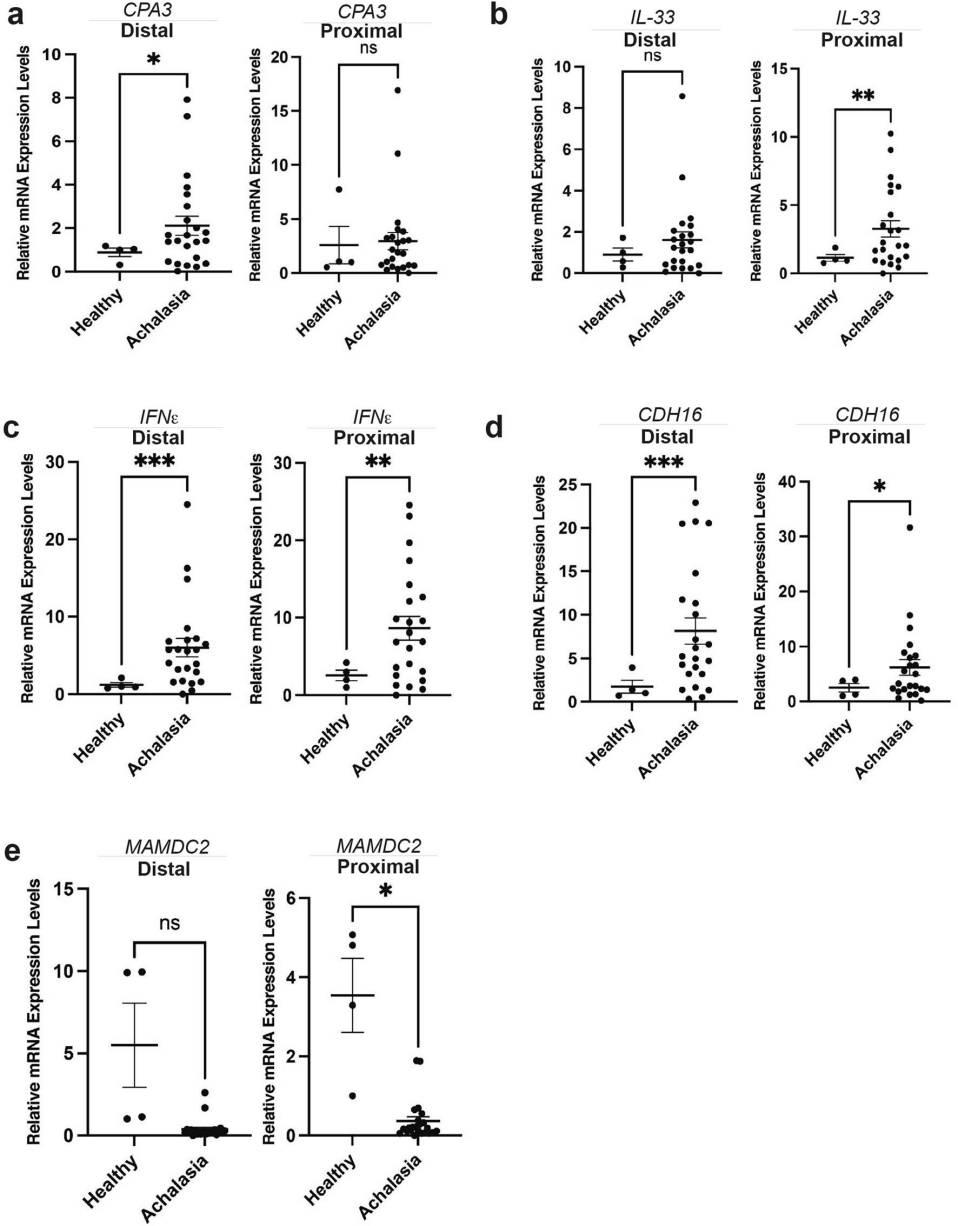
Using qPCR, mRNA expression levels of DEGs were determined in a validation cohort of achalasia. (**a**) *CPA3* is enriched in the esophageal mucosa of achalasia subjects in distal esophagus. *P < 0.02. (**b**) *IL-33* mRNA expression levels are increased in the proximal esophagus of achalasia subjects. **P < 0.005. (**c**) Increased *IFNε* is seen in both proximal and distal esophageal achalasia. **P < 0.002, ***P < 0.001. (**d**) The proximal and distal esophageal mucosa of achalasia is enriched for *CDH16*. *P < 0.05, ***P < 0.001. (**e**) Decreased *MAMDC2* is observed in the proximal esophageal mucosa of achalasia. *P < 0.05. n = 4 healthy control and 23 achalasia.

To validate the DEGs identified by RNA-sequencing, we performed quantitative PCR (qPCR) on esophageal mucosal samples in a distinct cohort from that used for RNA-seq (4 controls, 23 achalasia patients). Table 1 shows the clinical characteristic of the enrolled subjects. Among the genes identified by RNA-sequencing, we selected 5 genes to be validated by qPCR: the mast cell specific protease *carboxypeptidase* (*CPA3*), the cytokine *interleukin 33* (*IL-33*), the type 1 interferon family member *interferon epsilon* (*IFNε*), *the antiviral response gene MAM domain containing 2* (*MAMDC2*) and the cell adhesion molecule *cadherin 16* (*CHD16*). As shown in Fig. 5a, CPA3 mRNA expression levels were significantly up-regulated in the distal mucosa of achalasia subjects compared to controls, but not in the proximal esophagus. On the other hand, the cytokine IL-33, known to activate target cells such as mast cells and type 2 innate lymphoid cells^15^, had significant increased mRNA expression levels in the proximal esophageal mucosa of achalasia patients, but this change was not significant in the distal esophagus (Fig. 5b). We also observed that achalasia patients had a strong increase in mRNA expression levels of the type I interferon *IFNε,* in both the distal and proximal esophageal mucosa compared to controls (Fig. 5c). Interestingly, a significant increase in *CDH16*, an atypical member of the cadherin family^16^, was seen in both the proximal and distal esophageal mucosa of achalasia subjects compared to controls (Fig. 5d). Finally, we observed a significant decrease in *MAMDC2* in the proximal esophageal mucosa of achalasia patients compared to controls, but this change was not significant in the distal esophagus (Fig. 5e).

### Infiltration of intraepithelial leukocytes is detected in the esophageal epithelium of achalasia patients

**Figure 6:**
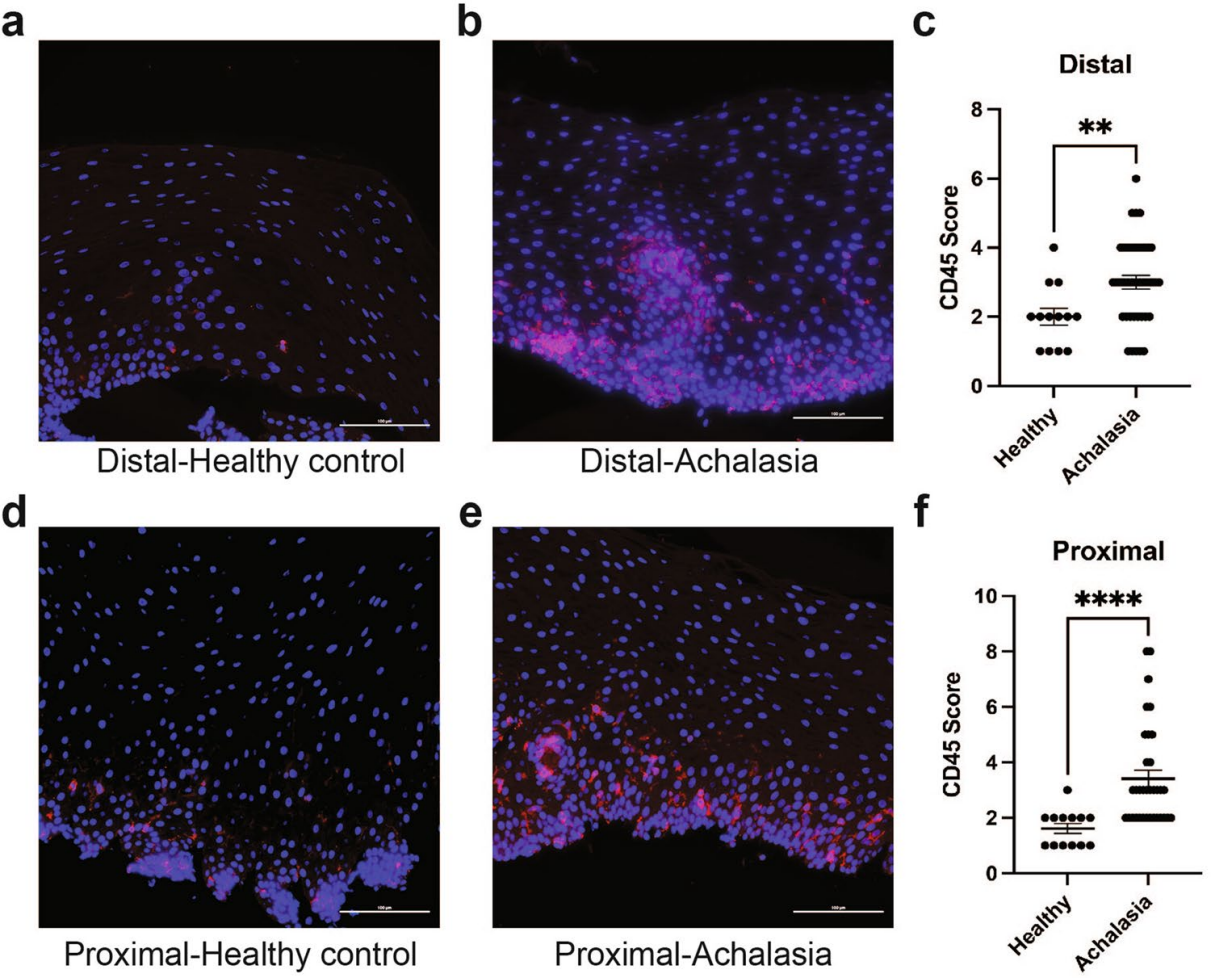
Increase infiltration of intraepithelial leukocyte in achalasia. (**a**,**b**,**d**,**e**) Representative immunofluorescence for the leukocyte marker CD45 (red) in the esophageal mucosa of achalasia subjects (**b**,**e**) compared to healthy control (**a**,**d**). Results from distal esophagus are shown in (**a**,**b**) as well as results from proximal esophagus are shown in (**d**,**e**). Dapi (blue) was used as a nuclear stain. Scale bars: 100 μm. (**c**,**f**) Column graph with individual values showing scoring for CD45+ intraepithelial leukocytes in distal (**c**) and proximal (**f**) esophageal mucosa of achalasia subjects compared to healthy controls. **P < 0.005, ****P < 0.0001. n = 12 healthy controls and 38 achalasia.

Our RNA-seq and qPCR analyses showed changes in many genes associated with inflammation and immune cell infiltration in achalasia. We first examined the presence of immune cells by performing immunofluorescence to detect the leukocyte marker CD45 in human esophageal mucosal biopsies from achalasia and healthy subjects. As shown in Fig. 6, immunostaining and scoring for CD45 showed increased infiltration of intraepithelial leukocytes in the distal esophageal mucosa of achalasia subjects (Fig. 6b,c), compared to controls (Fig. 6a,c). A significant increase in the recruitment of intraepithelial leukocytes was also observed in the proximal esophageal mucosa of achalasia patients (Fig. 6e,f) compared to controls (Fig. 6d,f).

### Distinct changes in gene expression and differentially regulated pathways are observed in the proximal and distal esophagus of type I versus type II achalasia patients

**Figure 7:**
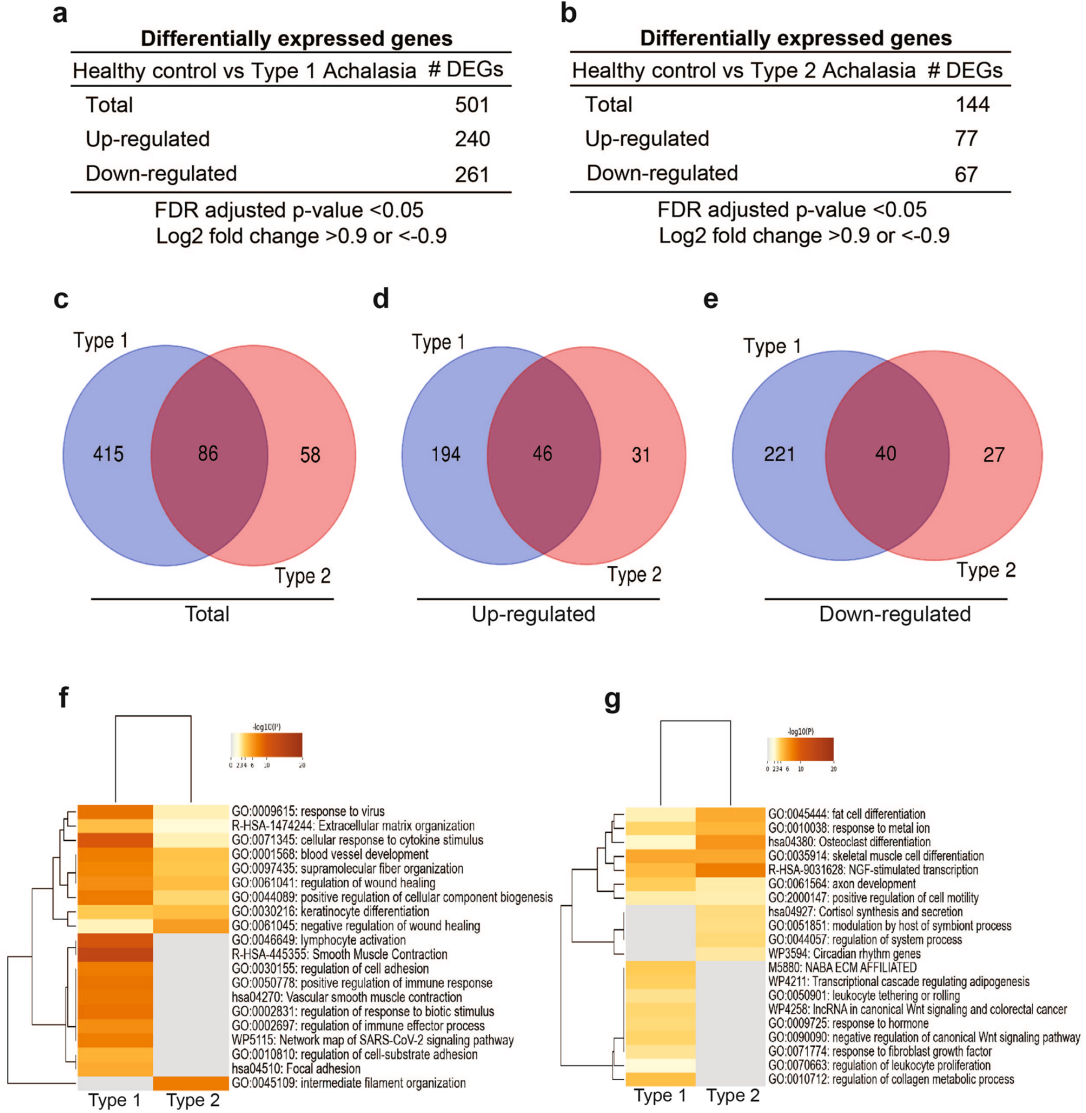
Differential gene expression analysis comparing changes in distal esophageal mucosa of type I and/or type II achalasia versus controls. (**a**) The number of differentially expressed genes (DEGs) in type I achalasia versus heathy controls based on a log2 fold change of 0.9 and FDR < 0.05. (**b**) Differentially expressed genes between type II achalasia and healthy controls were selected based on a log2 fold change of 0.9 and FDR < 0.05. (**c**) Venn diagram showing overlap of total DEGs in type I and type II achalasia subjects. (**d**) Venn diagram showing overlap of up-regulated DEGs between type I and type II achalasia subjects. (**e**) The overlap of down-regulated DEGs between type I and type II achalasia subjects is shown in a Venn diagram. (**f**,**g**) DEGs from each comparison were used to identify enrichment of functional pathways by Gene Ontology analysis. (**f**) A heatmap of the top up-regulated pathways is shown. (**g**) A heatmap of the top down-regulated pathways is shown.

**Figure 8:**
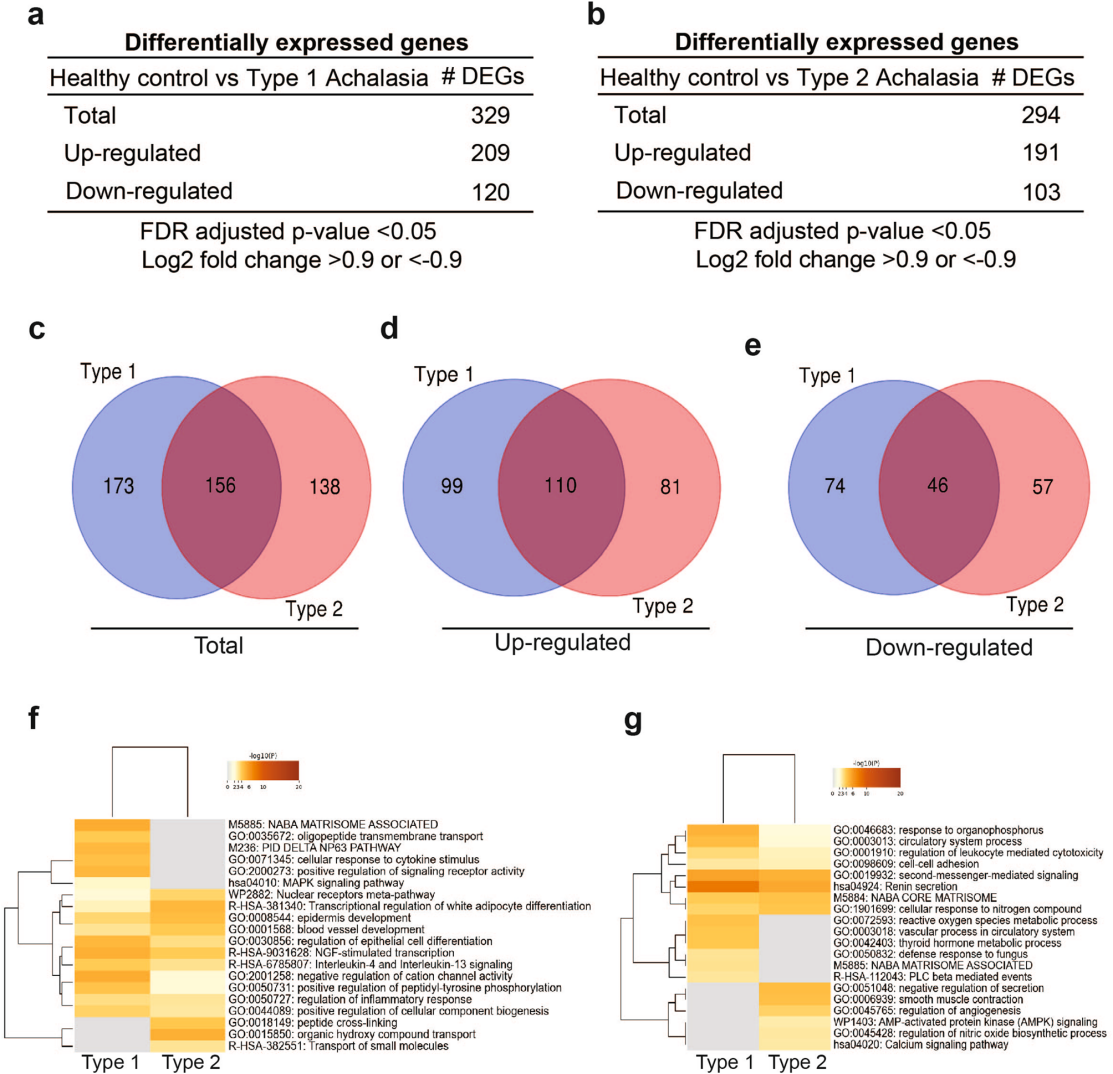
Comparison of differential gene expression analysis in proximal esophageal mucosa of type I and/or type II achalasia compared to healthy controls. (**a**,**b**) Differentially expressed genes in type I achalasia (**a**) or type II achalasia (**b**) versus healthy controls were selected based on a log2 fold change of 0.9 and FDR < 0.05. (**c**–**e**) Venn diagram showing overlap of DEGs in type I and type II achalasia subjects. (**c**) Total DEG is shown. (**d**) Up-regulated DEGs between type I and type II achalasia are illustrated. (**e**) Down-regulated DEGs between type I and type II achalasia are shown. (**f**,**g**) Gene ontology analysis using Metascape. (**f**) A heatmap of the top up-regulated pathways is shown. (**g**) A heatmap of the top down-regulated pathways is shown.

Given that type I achalasia is usually a later phase of disease progression, we next determined differential gene expression between type I or type II achalasia and healthy controls, in both proximal and distal esophagus. As showed in Fig. 7a, we identified 501 DEGs (240 up-regulated and 261 down-regulated) in the distal esophagus of type I achalasia compared to healthy controls. For type II achalasia, we found 144 DEGs in distal esophagus; 77 of these DEGs were up-regulated and 67 DEGs were down-regulated (Fig. 7b). In the proximal esophagus of type 1 achalasia, we identified 329 DEGs (209 up-regulated and 120 down-regulated) (Fig. 8a). On the other hand, the type II achalasia had 294 DEGs (191 up-regulated and 103 down-regulated) in proximal esophagus (Fig. 8b). We then determined the number of DEGs that were common or exclusive to type I and type II achalasia. In distal esophagus, we found 86 DEGs to be common between type I and type II achalasia (Fig. 7c). A total of 415 DEGs were exclusive to type I achalasia (Fig. 7c): 194 DEGs were up-regulated (Fig. 7d) and 221 were down-regulated (Fig. 7e). We found 58 DEGs to be exclusive to type II achalasia (Fig. 7c). Of these 58 genes, 31 were up-regulated (Fig. 7d) and 27 were down-regulated (Fig. 7e). In proximal esophagus, a total of 156 DEGs were common to type I and type II achalasia (Fig. 8c): 110 were up-regulated (Fig. 8d) and 46 were down-regulated (Fig. 8e). A total of 173 DEGs were exclusive to type I achalasia (Fig. 8c). 99 of those genes were up-regulated (Fig. 8b) and 74 were down-regulated (Fig. 8e). Type II achalasia had a total of 138 DEGs (Fig. 8c): 81 genes were up-regulated (Fig. 8d) and 57 genes were down-regulated (Fig. 8e).

Lists of DEGs from type I achalasia and type II achalasia were used to perform Gene Set Enrichment Analysis using Metascape (Figs. 7f,g, 8f,g) and GSEA (Supplementary Figs. 3–6). As shown in Fig. 7f, pathways associated to lymphocyte activation, regulation of cell adhesion, smooth muscle contraction, positive regulation of immune response and focal adhesion were enriched in the distal esophagus of type 1 achalasia. We also found decreased gene regulation of genes associated to pathways related to extracellular matrix, leukocyte tethering or rolling, negative regulation of canonical Wnt signaling and regulation of leukocyte proliferation (Fig. 7g). In the distal esophagus of type II achalasia, we observed increased gene regulation in pathways related to intermediate filament organization and negative regulation of wound healing (Fig. 7f) and decreased expression of genes related to pathways such as fat cell differentiation, NGF stimulated transcription and regulation of system process (Fig. 7g). Analyses performed in proximal esophagus showed the enrichment of pathways related to matrisome, delta Np63 pathway, positive regulation of signaling receptor activity in type I achalasia (Fig. 8f). Decreased gene regulation of genes associated to reactive oxygen species metabolic process, defense response to fungus, matrisome associated and PLC beta mediated events was seen (Fig. 8g). In type II achalasia, increased expression of genes related to peptide cross-linking, organic hydroxy compound transport and transport of small molecules was observed (Fig. 8f). Analyses also showed decreased gene regulation of genes associated to pathways related to negative regulation of secretion, smooth muscle contraction and regulation of angiogenesis (Fig. 8g).

## Discussion

Over the past decades, transcriptome profiling has been a common approach used to characterize the molecular changes occurring in human disease, which has led to the identification of numerous molecular biomarkers and new therapeutic targets. This is the first study to evaluate transcriptomic changes in the esophageal mucosa of achalasia patients compared to healthy controls. We identified 65 DEGs in the distal esophageal mucosa and 120 DEGs in the proximal esophageal mucosa of achalasia patients. Among the differentially expressed genes, we identified distinct changes in cellular response induced by cytokine stimulus in distal esophageal achalasia biopsies. These findings are consistent with our knowledge of the pathophysiology of achalasia and its connection to inflammation. Persistent esophageal distension leads to food stasis and liquid residue that sometimes ferments causing bacterial overgrowth. Over time this predisposes to chronic inflammation and increases the risk of developing esophageal squamous cell cancer^3,4^. Furthermore, our study confirms the presence of esophagitis previously reported in histological studies of the esophageal mucosa in achalasia^17–19^, which we confirmed in our study as well.

Among the inflammation-related genes, our study indicates significant up-regulation of the mast cell peptidase CPA3 in the mucosa of achalasia patients. Interestingly, previous studies showed significant mast cell infiltration or a change in their distribution and degranulation in the LES muscle of achalasia patients compared to controls^20–22^. However, the significance of mast cell degranulation and infiltration in the LES of achalasia patients, as well as the function of intraepithelial mast cells compared to LES mast cells needs to be determined. Nonetheless, this suggests a potential role for mast cells in the pathogenesis of achalasia. Further supporting this idea, we observed increased expression of the cytokine *IL-33*, an alarmin generally expressed by esophageal epithelial cells, fibroblasts and endothelial cells^23^. IL-33 is released extracellularly upon stimulus and can activate mast cells^24^, eosinophils^25^, basophils^26^ and type 2 innate lymphoid cells^27^. IL-33 plays a role in sensing damage caused by inflammation and is reportedly important in the pathogenesis of eosinophilic esophagitis^28,29^ and reflux esophagitis^30,31^. Although our findings suggest an association between CPA3+ mast cells and IL-33 in the esophageal mucosa of achalasia, the lack of significant increase of *CPA3* in proximal esophagus also implies a role for IL-33 on other immune cell types in achalasia.

Our transcriptomic analyses also showed that genes associated with the defense response to virus are enriched in the distal esophageal mucosa of achalasia patients. Interestingly, a possible association between achalasia, the destruction of myenteric neurons and viral infection has been proposed, indicating a possible association between achalasia and viral infection. Potential viruses associated with achalasia include herpes simplex virus (HSV)-1, varicella-zoster virus (VZV), measles, mumps, and human immunodeficiency virus (HIV)^32^. Among the up-regulated genes associated to viral response was *IFNε*, a unique member of the type I interferon family which plays crucial role in innate and adaptive responses to viral infection^33^. In the lung mucosa, IFNε induced the infiltration of a functional/cytotoxic CD8+CD4+ T cell population subset^34^. In the esophageal mucosa of achalasia patients, CD4+ T cells are shown to be a predominant immune cell population^17,19^. The function of IFNε, and its impact on intraepithelial leukocyte activation in the esophagus and in achalasia is yet to be determined.

Significantly, our findings show molecular heterogeneity between proximal and distal mucosa in the esophagus of achalasia patients, with few shared genes that are differentially expressed when compared to healthy subjects. Thinking about the pathophysiology of achalasia, this heterogeneity makes sense. During disease progression, achalasia patients may develop massive dilatation of the esophageal body, subjecting the esophageal mucosa to significant mechanical stress. The distal esophageal mucosa gets more exposure to food stasis as the result of gravity. Given that the primary etiology of achalasia involves the lower esophageal sphincter and distal esophagus, studies characterizing changes in the esophageal mucosa in achalasia patients have focused mostly on the distal esophageal mucosa. However, our findings suggest that including separate analyses of the proximal esophageal mucosa could offer additional insights.

Our study also demonstrates transcriptomic differences between type I and type II achalasia, both in proximal and distal esophagus. Given that type I achalasia is believed to be a more advanced stage of achalasia compared to type II, with the presence of food stasis and dilatation^6^, it is not surprising that we observed a higher number of DEGs in type I achalasia compared to type II, when both compared to healthy controls. These changes in DEGs in type I achalasia also correlate with the enrichment of pathways associated to wound healing and the regulation of immune response. Furthermore, the gradual increase in a subset of genes and pathways from type II to type I achalasia strongly supports at the molecular level the concept of type I achalasia representing a progression of the disease from type II achalasia. These currents results are based on our current cohort of 4 type I achalasia patients and 9 type II achalasia patients and give strong support to continue investigating the molecular differences between type I and type II achalasia in a larger cohort, which will allow greater power to directly uncover the changes that occur in the progression from type I to type II achalasia.

One major limitation of studies involving patients with early-stage achalasia is the acquisition of esophageal biopsies, which are often limited to the epithelium. Furthermore, we have observed increased epithelial thickness in most achalasia subjects, resulting in partial absence of the basal cell layer in some of these biopsies, while others have presence of some lamina propria. This can introduce variability in gene expression. Furthermore, given the etiology of achalasia, performing analyses on LES and muscle biopsies in parallel with esophageal mucosal samples from the same patients would be extremely valuable and informative. Unfortunately, acquiring muscle tissue from achalasia subjects is usually done during myotomy surgery, thus limiting this option. Although bulk RNA-sequencing was helpful in providing an overview of gene expression analyses in the esophageal mucosa of type I and type II achalasia, it reflects the average gene expression across thousands of cells. Therefore, it is possible that cellular heterogeneity in the disease could be masked. Thus, combining our bulk transcriptomic findings with single cell transcriptomic analyses in the future could provide additional insights into the pathogenesis of achalasia.

In conclusion, our study demonstrated transcriptomic differences in the esophageal mucosa in type I and type II achalasia compared to control subjects. We also reported regional differences in the distal vs proximal esophageal mucosa of achalasia subjects.

## Methods

### Subject sample collection and processing

Thirty-seven patients with newly diagnosed type 1 or type 2 achalasia according to the Chicago Classification of esophageal motility disorders v4.0^35^ encountered at the Esophageal Center at Northwestern were included. Type 3 achalasia patients were excluded because the spastic contractions in type 3 achalasia have unique physiology independent of bolus retention and sphincter function. Patients were also excluded if they had previously been treated for achalasia with pneumatic dilation, Heller’s myotomy, or PerOral Endoscopic Myotomy (POEM). All achalasia patients underwent endoscopy, HRM, barium esophagram, and functional luminal imaging probe (FLIP) Panometry. A subject’s esophagus was considered dilated when the esophageal width measured ≥ 5 cm on barium esophagram. All procedures using human tissue received approval from the Northwestern Institutional Review Board (STU00208111) and all methods were performed in accordance with the relevant guidelines and regulations. Informed consent was obtained from all subjects/legal guardians.

Healthy, asymptomatic (ie, free of esophageal symptoms including dysphagia, heartburn, and chest pain), adult volunteers, “controls”, were enrolled. Potential subjects were excluded for a previous diagnosis of esophageal, autoimmune, or eating disorders. Additional exclusion criteria included use of antacids or proton pump inhibitors, body mass index greater than 30 kg/m^2^, or a history of tobacco use or alcohol abuse. The controls underwent endoscopy, HRM, and FLIP, as previously described^36,37^.

For both patients and controls, esophageal mucosal biopsies were collected during sedated endoscopy through the Digestive Health Foundation Biorepository. Biopsies were collected from the distal and proximal esophagus, at 5 and 15 cm above the squamocolumnar junction, respectively. Briefly, for histology and immunostaining studies, tissue was fixed in neutral buffered formalin (Fisher Scientific, Hampton, NH) for 24 h, embedded in paraffin, and 4-μm sections were applied to positively charged slides. Slides were stained with hematoxylin and eosin, and images were captured on a Nikon Eclipse Ci-E microscope with a Nikon DS-Ri2 camera and NIS Elements software. For RNA studies, tissue was stored in RNA later and AllProtect (Thermo Fisher Scientific, Pittsburgh, PA) and stored at − 80 °C until RNA extraction.

### RNA isolation and quantitative PCR

Esophageal mucosal biopsies were homogenized prior to RNA extraction in RLT lysis buffer (Qiagen). Total RNA was extracted using the RNeasy kit according to the manufacturer’s instructions (Qiagen, Germantown, MD). The Maxima First-Strand complementary DNA Synthesis for Reverse-Transcription quantitative PCR kit (Thermo Fisher Scientific) was used for reverse transcription. Quantitative real-time PCR was performed using TaqMan Universal Master Mix (Thermo Fisher Scientific). TATA box binding protein or GAPDH genes were used as the internal control.

### Bulk RNA-sequencing

cDNA libraries were generated using the Tru-Seq Stranded mRNA-seq library prep (Illumina, San Diego, CA) following the manufacturer’s instructions. Sequencing was performed using Illumina HiSeq 4000 (Northwestern University NuSeq Core Facility). The quality of DNA reads, in fastq format, was evaluated using FastQC. Adapters were trimmed and reads of poor quality or aligning to rRNA sequences were filtered. The cleaned reads were aligned to the *Homo sapiens* genome (hg38) using STAR^38^. In conjunction with a gene annotation file for hg38 obtained from UCSC (University of California Santa Cruz; http://genome.ucsc.edu), read counts for each gene were calculated using HTSeq-count^39^. DESeq2 was used to determine normalization and differential expression^40^. The cutoff for determining significantly differentially expressed genes was an FDR-adjusted p-value less than 0.05. RNA-seq was deposited in Gene Expression Omnibus (GEO #201699) and can be accessed at http://www.ncbi.nlm.nih.gov/geo/query/acc.cgi?acc=GSE201699. A multivariate principal component analysis (PCA) was carried out on the entire dataset to reduce data dimensionality and to assess clustering. Gene ontology (GO) terms were identified using Metascape online platform (http://metascape.org)^41^ and GSEA (https://www.gsea-msigdb.org/gsea/index.jsp)^42,43^.

### Immunohistochemistry and immunofluorescence

Heat antigen retrieval (2100 Antigen Retriever, Electron Microscopy Sciences, Hatfield, PA) was performed for paraffin-embedded esophageal sections and slides were incubated with the following antibodies: 1:500 rabbit anti-CD45 (AB10558, Abcam, Cambridge, MA), 1:500 rabbit anti-CPA3 (HPA008689, Sigma). Species-specific secondary antibodies were added, and detection was performed as previously described^44^. For fluorescent labeling, Alexa Fluor™ 488 (#A32814, Thermo Fisher Scientific) was used. Dapi was used as a nuclear stain. CD45 staining was scored based on (1) the percent surface area covered by positive cells on a scale from 0 to 4 (0 = none, 1 = 1–25%, 2 = 26–50%, 3 = 51–75%, 4 = 76–100%) and (2) localization of positive cells in the epithelium on a scale from 1 to 4 (1 = localized around the papilla, 2 = slightly away from papilla, 3 = away from papilla and at another location in epithelium, 4 = throughout the epithelium). Scoring for CPA3 was done by counting the number of positive cells per high power field. Scoring was done by two blinded investigators. Images were captured on a Nikon Eclipse Ci-E microscope with a Nikon DS-Ri2 camera and NIS Elements software.

### Statistical analyses

Results are expressed as mean ± SEM, with statistical differences performed on normalized values between experimental conditions established at 95% confidence. The Welch’s t-test was used to indicate statistical difference between groups. All statistics were performed using Graph Pad Prism version 9.2.0 (Graph Pad software, San Diego, CA).

## Supplementary Information

Supplementary Figures.

Supplementary Tables.

## Data Availability

RNA-seq was deposited in Gene Expression Omnibus (GEO #201699) and can be accessed at http://www.ncbi.nlm.nih.gov/geo/query/acc.cgi?acc=GSE201699.
