# Supplementary material for: RNA-sequencing reveals molecular and regional differences in the esophageal mucosa of achalasia patients
Source: Sci Rep. 2022 Nov 30;12:20616. doi: 10.1038/s41598-022-25103-7 (PMC9712691; doi:10.1038/s41598-022-25103-7)
Supplement: Supplementary file 1 — Supplementary Figures. [file 41598_2022_25103_MOESM1_ESM.docx]

**Supplementary Figure 1.** **Gene Set Enrichment Analysis (GSEA) for gene ontology (GO) between achalasia and healthy controls in distal esophagus.** The top terms of biological processes in GO enrichment analysis are shown.

**Supplementary Figure 2. Gene Set Enrichment Analysis (GSEA) for gene ontology (GO) between achalasia and healthy controls in proximal esophagus.** The top terms of biological processes in GO enrichment analysis are shown.

**Supplementary Figure 3. Gene Set Enrichment Analysis (GSEA) for gene ontology (GO) between type I achalasia and healthy controls in distal esophagus.** The top terms of biological processes in GO enrichment analysis are shown.

**Supplementary Figure 4. Gene Set Enrichment Analysis (GSEA) for gene ontology (GO) between type II achalasia and healthy controls in distal esophagus.** The top terms of biological processes in GO enrichment analysis are shown.

**Supplementary Figure 5. Gene Set Enrichment Analysis (GSEA) for gene ontology (GO) between type I achalasia and healthy controls in proximal esophagus.** The top terms of biological processes in GO enrichment analysis are shown.

**Supplementary Figure 6. Gene Set Enrichment Analysis (GSEA) for gene ontology (GO) between type II achalasia and healthy controls in proximal esophagus.** The top terms of biological processes in GO enrichment analysis are shown.
