# Supplementary material for: RNA-sequencing reveals molecular and regional differences in the esophageal mucosa of achalasia patients
Source: Sci Rep. 2022 Nov 30;12:20616. doi: 10.1038/s41598-022-25103-7 (PMC9712691; doi:10.1038/s41598-022-25103-7)
Supplement: Supplementary file 2 — Supplementary Tables. [file 41598_2022_25103_MOESM2_ESM.docx]

**Supplementary Table 1. Genes differentially expressed in distal esophagus of achalasia vs. healthy control**

| **Gene Symbol** | **Log2 Fold Change** | **FDR Adj p Value** | **Description** |
| --- | --- | --- | --- |
| TPPP3 | -1.59421207 | 5.96E-08 | tubulin polymerization promoting protein family member 3 |
| FOSB | -1.498044227 | 5.96E-08 | FosB proto-oncogene, AP-1 transcription factor subunit |
| LRMP | -1.39651227 | 5.31E-07 | lymphoid restricted membrane protein |
| FCGR2B | -0.977355934 | 9.08E-07 | Fc fragment of IgG receptor IIb |
| JUN | -1.118405973 | 9.65E-07 | Jun proto-oncogene, AP-1 transcription factor subunit |
| SORCS2 | 1.085147946 | 1.06E-06 | sortilin related VPS10 domain containing receptor 2 |
| DZIP1 | 1.164566789 | 1.27E-06 | DAZ interacting zinc finger protein 1 |
| IFNE | 1.395468185 | 1.48E-06 | interferon epsilon |
| PHLDB2 | 1.31752458 | 2.49E-06 | pleckstrin homology like domain family B member 2 |
| MAMDC2 | -1.313791571 | 2.78E-06 | MAM domain containing 2 |
| CLEC4F | -1.148203713 | 6.97E-06 | C-type lectin domain family 4 member F |
| BASP1 | -1.061973298 | 9.45E-06 | brain abundant membrane attached signal protein 1 |
| CAPN6 | 1.165873042 | 1.31E-05 | calpain 6 |
| SLITRK5 | -1.28270121 | 1.36E-05 | SLIT and NTRK like family member 5 |
| FOS | -1.213095866 | 1.47E-05 | Fos proto-oncogene, AP-1 transcription factor subunit |
| PTPRD | 1.180777967 | 2.14E-05 | protein tyrosine phosphatase, receptor type D |
| ARHGAP39 | -1.02482203 | 2.28E-05 | Rho GTPase activating protein 39 |
| MIR5572 | 1.005934748 | 2.33E-05 | microRNA 5572 |
| ARNT2 | 1.005934748 | 2.33E-05 | aryl hydrocarbon receptor nuclear translocator 2 |
| LYPD2 | -1.124623378 | 2.79E-05 | LY6/PLAUR domain containing 2 |
| RTN1 | -0.938134533 | 5.88E-05 | reticulon 1 |
| CRIP1 | -1.166545557 | 7.13E-05 | cysteine rich protein 1 |
| MARCO | 0.992191253 | 8.33E-05 | macrophage receptor with collagenous structure |
| LOC100506885 | -1.036222869 | 0.000113534 | uncharacterized LOC100506885 |
| CX3CR1 | -1.036820073 | 0.000115941 | C-X3-C motif chemokine receptor 1 |
| PLA2G4E | 1.1648397 | 0.000115941 | phospholipase A2 group IVE |
| C8orf82 | -1.124693424 | 0.000115941 | chromosome 8 open reading frame 82 |
| DNAJB5 | 1.017533736 | 0.000115941 | DnaJ heat shock protein family (Hsp40) member B5 |
| LIPK | 1.142615327 | 0.000125007 | lipase family member K |
| SLC15A1 | 1.083967503 | 0.000130769 | solute carrier family 15 member 1 |
| OXGR1 | -1.148011673 | 0.000131796 | oxoglutarate receptor 1 |
| LAMB4 | -1.143252194 | 0.000139241 | laminin subunit beta 4 |
| ADTRP | 0.970282734 | 0.00018804 | androgen dependent TFPI regulating protein |
| DDIT4 | 1.077708186 | 0.000211714 | DNA damage inducible transcript 4 |
| LINC01587 | -1.031974766 | 0.00021843 | long intergenic non-protein coding RNA 1587 |
| MUC22 | -1.079005357 | 0.000259984 | mucin 22 |
| CARD18 | 1.085590796 | 0.000296696 | caspase recruitment domain family member 18 |
| HBA2 | -1.068324127 | 0.000304672 | hemoglobin subunit alpha 2 |
| P2RY13 | -0.900301872 | 0.000304672 | purinergic receptor P2Y13 |
| TNNT3 | -1.092500732 | 0.000331702 | troponin T3, fast skeletal type |
| LRRC14 | -1.055107014 | 0.000331702 | leucine rich repeat containing 14 |
| CRTAC1 | -1.09208727 | 0.000331702 | cartilage acidic protein 1 |
| MYO1B | 0.931285457 | 0.000387386 | myosin IB |
| RECQL4 | -1.055514538 | 0.00069208 | RecQ like helicase 4 |
| C1orf216 | 0.911722439 | 0.00069208 | chromosome 1 open reading frame 216 |
| GFPT2 | 1.045585229 | 0.000692607 | glutamine-fructose-6-phosphate transaminase 2 |
| IL33 | 0.999679407 | 0.000828754 | interleukin 33 |
| PSCA | -0.950946934 | 0.000874907 | prostate stem cell antigen |
| RGS20 | 0.940640604 | 0.001028076 | regulator of G-protein signaling 20 |
| LOX | 0.916775335 | 0.001055767 | lysyl oxidase |
| TMEM35A | 1.021770356 | 0.001160719 | transmembrane protein 35A |
| GNA14 | -0.915840607 | 0.001281901 | G protein subunit alpha 14 |
| GLDC | 1.012432825 | 0.001318609 | glycine decarboxylase |
| PRRX1 | 0.964689517 | 0.001454907 | paired related homeobox 1 |
| ATP13A5 | 0.968026664 | 0.001517636 | ATPase 13A5 |
| CPA3 | 0.96248142 | 0.001659822 | carboxypeptidase A3 |
| APOBEC3A | 0.958470262 | 0.001892124 | apolipoprotein B mRNA editing enzyme catalytic subunit 3A |
| APOBEC3A_B | 0.958470262 | 0.001892124 | APOBEC3A and APOBEC3B deletion hybrid |
| ATF3 | -0.969413 | 0.002258947 | activating transcription factor 3 |
| GEM | -0.972338405 | 0.002485346 | GTP binding protein overexpressed in skeletal muscle |
| PNLIPRP3 | 0.964627894 | 0.002751794 | pancreatic lipase related protein 3 |
| CREB5 | -0.932998588 | 0.002796821 | cAMP responsive element binding protein 5 |
| CRYM | -0.910761703 | 0.004033968 | crystallin mu |
| GPT | -0.91705433 | 0.004098961 | glutamic--pyruvic transaminase |

**Supplementary Table 2. Genes differentially expressed in distal esophagus of achalasia vs. healthy control**

| **Gene Symbol** | **Log2 Fold Change** | **FDR Adj p Value** | **Description** |
| --- | --- | --- | --- |
| TMEM238 | 0.965331516 | 3.16E-16 | transmembrane protein 238 |
| SLITRK5 | -2.253521423 | 8.31E-16 | SLIT and NTRK like family member 5 |
| DZIP1 | 1.329402794 | 6.59E-13 | DAZ interacting zinc finger protein 1 |
| MAMDC2 | -1.785088338 | 1.16E-12 | MAM domain containing 2 |
| DDIT4 | 1.429635517 | 6.87E-09 | DNA damage inducible transcript 4 |
| C17orf96 | 1.41140404 | 2.21E-08 | chromosome 17 open reading frame 96 |
| ALDOA | 1.160065279 | 1.21E-07 | aldolase, fructose-bisphosphate A |
| LRRC14 | -1.311667823 | 1.65E-07 | leucine rich repeat containing 14 |
| ID3 | 0.967600853 | 1.93E-07 | inhibitor of DNA binding 3, HLH protein |
| RECQL4 | -1.49817358 | 2.22E-07 | RecQ like helicase 4 |
| COL16A1 | 0.911073845 | 3.72E-07 | collagen type XVI alpha 1 chain |
| TPSAB1 | 1.42868489 | 4.45E-07 | tryptase alpha/beta 1 |
| FBXO2 | 0.914283793 | 4.82E-07 | F-box protein 2 |
| SLC2A12 | -1.112836914 | 5.49E-07 | solute carrier family 2 member 12 |
| ALCAM | -1.320780631 | 8.66E-07 | activated leukocyte cell adhesion molecule |
| HOMER3 | 1.011134169 | 1.04E-06 | homer scaffolding protein 3 |
| FABP12 | 1.172458712 | 1.84E-06 | fatty acid binding protein 12 |
| MIR4329 | -0.987827515 | 3.93E-06 | microRNA 4329 |
| AMOT | -0.987827515 | 3.93E-06 | angiomotin |
| C8orf82 | -0.96660123 | 4.31E-06 | chromosome 8 open reading frame 82 |
| C4orf48 | 1.324703739 | 4.32E-06 | chromosome 4 open reading frame 48 |
| RN7SL2 | 1.442206044 | 4.37E-06 | RNA, 7SL, cytoplasmic 2 |
| OXGR1 | -1.397897169 | 4.71E-06 | oxoglutarate receptor 1 |
| CTXN1 | 1.422153206 | 7.40E-06 | cortexin 1 |
| PGBD5 | 1.3127145 | 7.85E-06 | piggyBac transposable element derived 5 |
| HES5 | 1.191422971 | 1.05E-05 | hes family bHLH transcription factor 5 |
| DPYSL3 | -1.417979869 | 1.13E-05 | dihydropyrimidinase like 3 |
| TNFRSF12A | 1.177946242 | 1.13E-05 | TNF receptor superfamily member 12A |
| RRAD | 1.335361544 | 1.30E-05 | RRAD, Ras related glycolysis inhibitor and calcium channel regulator |
| LOC100506388 | 1.241198973 | 1.30E-05 | uncharacterized LOC100506388 |
| COLEC12 | -1.335792782 | 1.30E-05 | collectin subfamily member 12 |
| RPL39L | 0.927578835 | 1.95E-05 | ribosomal protein L39 like |
| FGF14 | -1.388095549 | 2.22E-05 | fibroblast growth factor 14 |
| TSPAN12 | -0.91170796 | 2.54E-05 | tetraspanin 12 |
| FRAS1 | -0.979750889 | 3.23E-05 | Fraser extracellular matrix complex subunit 1 |
| NRARP | 1.116036367 | 3.38E-05 | NOTCH-regulated ankyrin repeat protein |
| FMO9P | -1.312402519 | 4.00E-05 | flavin containing monooxygenase 9 pseudogene |
| TYMP | 1.00265148 | 5.29E-05 | thymidine phosphorylase |
| RNF152 | -0.911080138 | 6.55E-05 | ring finger protein 152 |
| IER5L | 0.998056829 | 6.59E-05 | immediate early response 5 like |
| C1orf216 | 1.110331247 | 7.17E-05 | chromosome 1 open reading frame 216 |
| MARCO | 1.326590795 | 7.21E-05 | macrophage receptor with collagenous structure |
| TMEM45A | 1.251723513 | 7.27E-05 | transmembrane protein 45A |
| PDGFD | -1.058158778 | 7.44E-05 | platelet derived growth factor D |
| ID1 | 1.01664509 | 7.85E-05 | inhibitor of DNA binding 1, HLH protein |
| ABHD8 | 1.007565321 | 7.85E-05 | abhydrolase domain containing 8 |
| EVA1B | 0.968355978 | 7.92E-05 | eva-1 homolog B |
| LRMP | -1.296017581 | 7.93E-05 | lymphoid restricted membrane protein |
| ARL2BP | 1.007330972 | 7.93E-05 | ADP ribosylation factor like GTPase 2 binding protein |
| MASP2 | -1.045869467 | 8.65E-05 | mannan binding lectin serine peptidase 2 |
| TNFRSF18 | 1.125517193 | 9.53E-05 | TNF receptor superfamily member 18 |
| PDXP | 0.940880998 | 0.000104654 | pyridoxal phosphatase |
| IRX4 | 1.001326491 | 0.000117231 | iroquois homeobox 4 |
| FSCN1 | 0.919905402 | 0.000117231 | fascin actin-bundling protein 1 |
| PDE1A | -1.133118119 | 0.000133494 | phosphodiesterase 1A |
| SHF | 1.096726956 | 0.000139744 | Src homology 2 domain containing F |
| SORCS2 | 1.069308986 | 0.000146444 | sortilin related VPS10 domain containing receptor 2 |
| PRRX2 | 1.158858567 | 0.000146444 | paired related homeobox 2 |
| CYP4F35P | -1.122308642 | 0.000150615 | cytochrome P450 family 4 subfamily F member 35, pseudogene |
| SBSPON | -1.107792735 | 0.00017873 | somatomedin B and thrombospondin type 1 domain containing |
| CDKN1C | 1.049349554 | 0.000183842 | cyclin dependent kinase inhibitor 1C |
| PRR7 | 0.92183342 | 0.000183853 | proline rich 7, synaptic |
| ICAM5 | 1.156957649 | 0.000230859 | intercellular adhesion molecule 5 |
| SLC26A2 | -1.009009164 | 0.000230859 | solute carrier family 26 member 2 |
| SLC15A1 | 1.0683038 | 0.000241015 | solute carrier family 15 member 1 |
| CAPN6 | 1.080329769 | 0.000241565 | calpain 6 |
| TMEM158 | 0.984283283 | 0.000251756 | transmembrane protein 158 (gene/pseudogene) |
| RASL12 | 1.090490781 | 0.000276688 | RAS like family 12 |
| CDH16 | 1.156557207 | 0.000305539 | cadherin 16 |
| SUSD2 | 1.005673908 | 0.000382291 | sushi domain containing 2 |
| OASL | 1.075848102 | 0.000442511 | 2'-5'-oligoadenylate synthetase like |
| CFTR | -0.938331508 | 0.000446303 | cystic fibrosis transmembrane conductance regulator |
| MIR6872 | 0.992030629 | 0.00046753 | microRNA 6872 |
| SEMA3B | 0.992030629 | 0.00046753 | semaphorin 3B |
| RNASE7 | -1.165055806 | 0.000683285 | ribonuclease A family member 7 |
| ZNF467 | 1.045625194 | 0.000698228 | zinc finger protein 467 |
| MMP17 | 1.033844836 | 0.000721457 | matrix metallopeptidase 17 |
| GFPT2 | 1.148786792 | 0.00075606 | glutamine-fructose-6-phosphate transaminase 2 |
| DNAJB5 | 0.911148273 | 0.000764529 | DnaJ heat shock protein family (Hsp40) member B5 |
| B3GAT2 | -0.923991088 | 0.000766886 | beta-1,3-glucuronyltransferase 2 |
| STXBP6 | -0.96446143 | 0.000808615 | syntaxin binding protein 6 |
| THBS2 | 1.04764342 | 0.000814632 | thrombospondin 2 |
| KREMEN2 | 0.988742394 | 0.00085748 | kringle containing transmembrane protein 2 |
| GCHFR | 1.012379545 | 0.000866151 | GTP cyclohydrolase I feedback regulator |
| GGT1 | 1.009745831 | 0.000898339 | gamma-glutamyltransferase 1 |
| GGTLC1 | 1.009745831 | 0.000898339 | gamma-glutamyltransferase light chain 1 |
| GGTLC2 | 1.009745831 | 0.000898339 | gamma-glutamyltransferase light chain 2 |
| CARD17 | 1.098288815 | 0.000923566 | caspase recruitment domain family member 17 |
| FAM131C | 1.084792941 | 0.001179737 | family with sequence similarity 131 member C |
| FAM25A | 1.04842954 | 0.001574831 | family with sequence similarity 25 member A |
| CARD18 | 1.000790025 | 0.001580053 | caspase recruitment domain family member 18 |
| CPA3 | 1.04014522 | 0.001998523 | carboxypeptidase A3 |
| MMP9 | 1.045395197 | 0.002180842 | matrix metallopeptidase 9 |
| SLC10A5 | -1.003580058 | 0.002200017 | solute carrier family 10 member 5 |
| LOC283140 | 0.927812332 | 0.002258585 | uncharacterized LOC283140 |
| ANK1 | 1.062977246 | 0.002319896 | ankyrin 1 |
| PNLIPRP3 | 1.04290138 | 0.00235623 | pancreatic lipase related protein 3 |
| PTGER1 | 0.968498135 | 0.002398759 | prostaglandin E receptor 1 |
| CLEC4F | -0.91738895 | 0.002450858 | C-type lectin domain family 4 member F |
| ANGPTL4 | 0.9843019 | 0.002598331 | angiopoietin like 4 |
| CHST6 | 0.909788058 | 0.002677795 | carbohydrate sulfotransferase 6 |
| TINAGL1 | 1.020831697 | 0.00300383 | tubulointerstitial nephritis antigen like 1 |
| PART1 | -0.972698538 | 0.003049141 | prostate androgen-regulated transcript 1 (non-protein coding) |
| CIB2 | 0.968267135 | 0.003097107 | calcium and integrin binding family member 2 |
| LOC102724344 | 1.026425486 | 0.00314207 | uncharacterized LOC102724344 |
| LRRC7 | -0.988748275 | 0.00322821 | leucine rich repeat containing 7 |
| IGFBP3 | -0.936728831 | 0.0036321 | insulin like growth factor binding protein 3 |
| CRYM | -0.997655406 | 0.003805815 | crystallin mu |
| FSTL4 | -0.930077768 | 0.003879363 | follistatin like 4 |
| MTUS2 | -0.918298795 | 0.004295572 | microtubule associated tumor suppressor candidate 2 |
| GLDC | 0.975368508 | 0.005213876 | glycine decarboxylase |
| TFAP2E | 0.942601886 | 0.005508216 | transcription factor AP-2 epsilon |
| TPSD1 | 0.973475562 | 0.005656311 | tryptase delta 1 |
| KIAA2022 | -0.912387235 | 0.006015828 | KIAA2022 |
| TPPP3 | -0.945254091 | 0.006317098 | tubulin polymerization promoting protein family member 3 |
| AGMAT | 0.904766363 | 0.006450561 | agmatinase |
| FETUB | -0.912616204 | 0.007023397 | fetuin B |
| PI3 | 0.940008656 | 0.008078682 | peptidase inhibitor 3 |
| CFD | 0.902578899 | 0.008629654 | complement factor D |

**Supplementary Table 3. Genes differentially expressed in distal esophagus of type 1 achalasia vs. healthy control**

| **Gene Symbol** | **Log2 Fold Change** | **FDR Adj p Value** | **Description** |
| --- | --- | --- | --- |
| DES | 11.9226757 | 0.00039159 | desmin |
| ACTG2 | 9.991699 | 3.66E-05 | actin gamma 2, smooth muscle |
| CHRDL1 | 7.24888243 | 0.00671687 | chordin like 1 |
| MYH11 | 6.71476279 | 7.80E-09 | myosin heavy chain 11 |
| GREM1 | 6.0107635 | 0.01667577 | gremlin 1, DAN family BMP antagonist |
| GABRB2 | 4.37458847 | 2.27E-06 | gamma-aminobutyric acid type A receptor subunit beta2 |
| TNFSF18 | 4.10540117 | 7.19E-08 | TNF superfamily member 18 |
| PRUNE2 | 4.10043354 | 0.00050937 | prune homolog 2 with BCH domain |
| LMOD1 | 4.09744636 | 0.00022549 | leiomodin 1 |
| MMP12 | 3.98888523 | 0.00985585 | matrix metallopeptidase 12 |
| NEXN | 3.93280001 | 0.00165166 | nexilin F-actin binding protein |
| PLA2G4E | 3.40323835 | 2.87E-19 | phospholipase A2 group IVE |
| APOBEC3A | 3.26025407 | 1.74E-10 | apolipoprotein B mRNA editing enzyme catalytic subunit 3A |
| APOBEC3A_B | 3.26025407 | 1.74E-10 | APOBEC3A and APOBEC3B deletion hybrid |
| LCE3A | 3.20967923 | 0.00609553 | late cornified envelope 3A |
| FN1 | 3.1317099 | 0.00430179 | fibronectin 1 |
| PGM5 | 2.94149357 | 0.00285265 | phosphoglucomutase 5 |
| WFDC12 | 2.88055735 | 0.00893969 | WAP four-disulfide core domain 12 |
| TAGLN | 2.83811525 | 0.00046191 | transgelin |
| KRT1 | 2.74071648 | 0.0038806 | keratin 1 |
| ACTA2 | 2.69207359 | 0.00084345 | actin alpha 2, smooth muscle |
| ARC | 2.66316054 | 0.0042092 | activity regulated cytoskeleton associated protein |
| MYL9 | 2.61662455 | 0.00430179 | myosin light chain 9 |
| LCE3E | 2.59734578 | 0.0097239 | late cornified envelope 3E |
| MMRN1 | 2.54823058 | 0.01902605 | multimerin 1 |
| FLNC | 2.53930607 | 0.0101001 | filamin C |
| PALM | 2.53291128 | 0.01361358 | paralemmin |
| GFPT2 | 2.52321952 | 0.00014369 | glutamine-fructose-6-phosphate transaminase 2 |
| PHLDB2 | 2.48224372 | 1.23E-14 | pleckstrin homology like domain family B member 2 |
| SYNPO2 | 2.47303282 | 2.43E-05 | synaptopodin 2 |
| CXCL10 | 2.38137348 | 6.86E-05 | C-X-C motif chemokine ligand 10 |
| SLC26A9 | 2.36837599 | 0.00592985 | solute carrier family 26 member 9 |
| MSRB3 | 2.24186426 | 0.0036905 | methionine sulfoxide reductase B3 |
| LINC02028 | 2.22748508 | 0.01181721 | long intergenic non-protein coding RNA 2028 |
| LIPK | 2.21716556 | 7.09E-10 | lipase family member K |
| GLDC | 2.19291787 | 0.00064221 | glycine decarboxylase |
| GSDMA | 2.17099674 | 0.00237877 | gasdermin A |
| PNLIPRP3 | 2.15266702 | 0.00170237 | pancreatic lipase related protein 3 |
| MYLK | 2.11957992 | 0.00823479 | myosin light chain kinase |
| PLA2G4D | 2.10350492 | 0.01534696 | phospholipase A2 group IVD |
| CPA3 | 2.07364391 | 0.00039715 | carboxypeptidase A3 |
| OASL | 2.04338144 | 0.00226275 | 2'-5'-oligoadenylate synthetase like |
| ATP13A5 | 2.03861583 | 4.60E-07 | ATPase 13A5 |
| ZFHX4 | 2.03487454 | 0.00670291 | zinc finger homeobox 4 |
| SLC15A1 | 2.01573081 | 0.00022477 | solute carrier family 15 member 1 |
| IL36G | 2.00484786 | 0.01154269 | interleukin 36 gamma |
| TMEM45A | 1.97251408 | 8.63E-05 | transmembrane protein 45A |
| HEPHL1 | 1.95073738 | 0.00055279 | hephaestin like 1 |
| LINC02026 | 1.92895486 | 0.00239744 | long intergenic non-protein coding RNA 2026 |
| CARD17 | 1.89614697 | 0.00426625 | caspase recruitment domain family member 17 |
| AKAP12 | 1.87411473 | 0.01916437 | A-kinase anchoring protein 12 |
| PRRX1 | 1.84861079 | 1.07E-07 | paired related homeobox 1 |
| PGBD5 | 1.84516925 | 0.01983432 | piggyBac transposable element derived 5 |
| MAP1B | 1.84191176 | 0.00075302 | microtubule associated protein 1B |
| BST2 | 1.82728638 | 0.04403291 | bone marrow stromal cell antigen 2 |
| IL7R | 1.80551434 | 0.03774518 | interleukin 7 receptor |
| LOX | 1.79415557 | 3.82E-11 | lysyl oxidase |
| HAL | 1.78689941 | 0.00013161 | histidine ammonia-lyase |
| TPM2 | 1.76711451 | 0.00213766 | tropomyosin 2 |
| CARD18 | 1.75485602 | 0.00162989 | caspase recruitment domain family member 18 |
| CAPN6 | 1.75261128 | 9.00E-07 | calpain 6 |
| KPRP | 1.74117521 | 0.02199091 | keratinocyte proline rich protein |
| IFI44 | 1.72504763 | 0.00094168 | interferon induced protein 44 |
| MS4A2 | 1.72436296 | 0.00231615 | membrane spanning 4-domains A2 |
| IL33 | 1.71064571 | 8.47E-06 | interleukin 33 |
| THEMIS | 1.68528873 | 0.01425917 | thymocyte selection associated |
| BMERB1 | 1.6795409 | 0.00029856 | bMERB domain containing 1 |
| MIAT | 1.64247394 | 0.00290233 | myocardial infarction associated transcript |
| PTPRD | 1.63935933 | 1.12E-07 | protein tyrosine phosphatase receptor type D |
| KLK6 | 1.62308213 | 0.00553854 | kallikrein related peptidase 6 |
| PTPRQ | 1.59660923 | 0.00313272 | protein tyrosine phosphatase receptor type Q |
| IFIT1 | 1.58740735 | 0.00284629 | interferon induced protein with tetratricopeptide repeats 1 |
| THBS2 | 1.57345009 | 3.95E-07 | thrombospondin 2 |
| RND1 | 1.56591762 | 0.04361893 | Rho family GTPase 1 |
| CDH17 | 1.56003976 | 0.00010291 | cadherin 17 |
| KLRD1 | 1.55828389 | 0.00910654 | killer cell lectin like receptor D1 |
| KLK7 | 1.55550431 | 0.00254185 | kallikrein related peptidase 7 |
| DDX60 | 1.54890109 | 3.52E-05 | DExD/H-box helicase 60 |
| DZIP1 | 1.53881544 | 3.20E-07 | DAZ interacting zinc finger protein 1 |
| GRAP2 | 1.51952832 | 0.00434394 | GRB2 related adaptor protein 2 |
| IKZF3 | 1.50608633 | 0.00043869 | IKAROS family zinc finger 3 |
| CXCL9 | 1.50401129 | 0.01671135 | C-X-C motif chemokine ligand 9 |
| FAM180A | 1.49441803 | 0.00226275 | family with sequence similarity 180 member A |
| SEMA3C | 1.4936082 | 4.68E-07 | semaphorin 3C |
| IDO1 | 1.48831989 | 0.04216379 | indoleamine 2,3-dioxygenase 1 |
| SUSD2 | 1.48455274 | 0.0083682 | sushi domain containing 2 |
| IFI44L | 1.47914941 | 0.04872641 | interferon induced protein 44 like |
| COL12A1 | 1.47826999 | 0.00423431 | collagen type XII alpha 1 chain |
| ARNT2 | 1.47295454 | 2.40E-05 | aryl hydrocarbon receptor nuclear translocator 2 |
| SYTL2 | 1.45666151 | 0.00254374 | synaptotagmin like 2 |
| ADTRP | 1.45158225 | 3.93E-06 | androgen dependent TFPI regulating protein |
| CD22 | 1.44995189 | 0.03989063 | CD22 molecule |
| TDRD6 | 1.44465932 | 0.00065638 | tudor domain containing 6 |
| RTP4 | 1.43889883 | 0.00096976 | receptor transporter protein 4 |
| GPR174 | 1.426837 | 0.00243223 | G protein-coupled receptor 174 |
| ALOXE3 | 1.42530512 | 0.02872075 | arachidonate lipoxygenase 3 |
| MARCO | 1.41777145 | 0.00059869 | macrophage receptor with collagenous structure |
| IL18RAP | 1.41234745 | 0.03350661 | interleukin 18 receptor accessory protein |
| GPR65 | 1.41080632 | 0.00599372 | G protein-coupled receptor 65 |
| ABCG1 | 1.38209196 | 0.00014048 | ATP binding cassette subfamily G member 1 |
| TPM1 | 1.36997198 | 0.00297516 | tropomyosin 1 |
| RUFY4 | 1.3694612 | 0.00012713 | RUN and FYVE domain containing 4 |
| STRA6 | 1.36693167 | 0.00013849 | signaling receptor and transporter of retinol STRA6 |
| LOC388282 | 1.36651458 | 0.00848572 | uncharacterized LOC388282 |
| PCAT2 | 1.36076734 | 1.42E-07 | prostate cancer associated transcript 2 |
| ITGA1 | 1.36041817 | 2.08E-05 | integrin subunit alpha 1 |
| DDIT4 | 1.35261891 | 0.00240379 | DNA damage inducible transcript 4 |
| CD36 | 1.3503907 | 0.00052944 | CD36 molecule |
| THSD7B | 1.34732916 | 0.00036421 | thrombospondin type 1 domain containing 7B |
| RGS20 | 1.33234548 | 0.00060428 | regulator of G protein signaling 20 |
| SORCS2 | 1.33042294 | 3.54E-06 | sortilin related VPS10 domain containing receptor 2 |
| ITK | 1.32657497 | 0.00335329 | IL2 inducible T cell kinase |
| CDH26 | 1.32310252 | 0.00761619 | cadherin 26 |
| ABCA12 | 1.32291648 | 0.00096943 | ATP binding cassette subfamily A member 12 |
| SLAMF6 | 1.31948014 | 0.01741984 | SLAM family member 6 |
| CLDN17 | 1.31689008 | 0.02395196 | claudin 17 |
| CEMIP | 1.31171734 | 1.48E-06 | cell migration inducing hyaluronidase 1 |
| CPA6 | 1.30999031 | 0.03498676 | carboxypeptidase A6 |
| CD28 | 1.30950912 | 0.00731528 | CD28 molecule |
| CHST6 | 1.3067278 | 0.01498964 | carbohydrate sulfotransferase 6 |
| RAP2C-AS1 | 1.30616598 | 0.00686491 | RAP2C antisense RNA 1 |
| HDC | 1.29835945 | 0.04016963 | histidine decarboxylase |
| CACNA1C-IT2 | 1.29450574 | 0.00435297 | CACNA1C intronic transcript 2 |
| CACNA1C | 1.29450574 | 0.00435297 | calcium voltage-gated channel subunit alpha1 C |
| SERPINB4 | 1.28913996 | 0.00326327 | serpin family B member 4 |
| AADACL2 | 1.26825361 | 0.04462876 | arylacetamide deacetylase like 2 |
| LOC100506497 | 1.26745911 | 0.00212848 | uncharacterized LOC100506497 |
| PPIAP46 | 1.24955317 | 0.02780557 | peptidylprolyl isomerase A pseudogene 46 |
| GGT1 | 1.24905239 | 0.00310904 | gamma-glutamyltransferase 1 |
| TESPA1 | 1.24494058 | 9.56E-05 | thymocyte expressed, positive selection associated 1 |
| THY1 | 1.24080508 | 0.02430307 | Thy-1 cell surface antigen |
| THSD7A | 1.24016098 | 0.01376723 | thrombospondin type 1 domain containing 7A |
| NLRC3 | 1.23423503 | 0.00235418 | NLR family CARD domain containing 3 |
| IQCA1 | 1.23242402 | 0.00717411 | IQ motif containing with AAA domain 1 |
| TFAP2E | 1.22767893 | 0.00745076 | transcription factor AP-2 epsilon |
| IFIT3 | 1.2247369 | 0.02353018 | interferon induced protein with tetratricopeptide repeats 3 |
| FMO3 | 1.22413625 | 0.02535416 | flavin containing dimethylaniline monoxygenase 3 |
| AMPD3 | 1.22366114 | 0.03537599 | adenosine monophosphate deaminase 3 |
| BIRC3 | 1.22350454 | 1.02E-05 | baculoviral IAP repeat containing 3 |
| FBXO39 | 1.22105101 | 0.00964193 | F-box protein 39 |
| WNT10B | 1.21612546 | 5.26E-07 | Wnt family member 10B |
| FABP12 | 1.21232817 | 0.00036842 | fatty acid binding protein 12 |
| NXPH3 | 1.20464919 | 0.00112585 | neurexophilin 3 |
| LRRC20 | 1.19958218 | 0.02070438 | leucine rich repeat containing 20 |
| CALD1 | 1.19907293 | 1.29E-06 | caldesmon 1 |
| NBEA | 1.19340638 | 0.04984551 | neurobeachin |
| FERMT2 | 1.18742824 | 0.03289379 | FERM domain containing kindlin 2 |
| COL6A3 | 1.18503171 | 0.02724716 | collagen type VI alpha 3 chain |
| GTF2H2C | 1.18345906 | 0.01153338 | GTF2H2 family member C |
| GTF2H2 | 1.18345906 | 0.01153338 | general transcription factor IIH subunit 2 |
| PARP15 | 1.18228414 | 0.01002954 | poly(ADP-ribose) polymerase family member 15 |
| QPCT | 1.17793499 | 0.00186702 | glutaminyl-peptide cyclotransferase |
| CCND2 | 1.17473101 | 1.51E-06 | cyclin D2 |
| GRPEL2 | 1.17457733 | 0.02353018 | GrpE like 2, mitochondrial |
| EPSTI1 | 1.17301477 | 0.00691193 | epithelial stromal interaction 1 |
| DDR2 | 1.17079967 | 0.02952789 | discoidin domain receptor tyrosine kinase 2 |
| C1orf216 | 1.15358692 | 0.00505499 | chromosome 1 open reading frame 216 |
| CEP19 | 1.14508048 | 0.0022 | centrosomal protein 19 |
| SAMSN1 | 1.13762494 | 0.00479428 | SAM domain, SH3 domain and nuclear localization signals 1 |
| KLHL6 | 1.1346619 | 0.01482516 | kelch like family member 6 |
| GABRA4 | 1.13444208 | 0.00249014 | gamma-aminobutyric acid type A receptor subunit alpha4 |
| ST8SIA1 | 1.12933359 | 0.00364188 | ST8 alpha-N-acetyl-neuraminide alpha-2,8-sialyltransferase 1 |
| SLC7A2 | 1.12765914 | 1.18E-05 | solute carrier family 7 member 2 |
| DDX60L | 1.11902958 | 1.00E-04 | DExD/H-box 60 like |
| TRIM22 | 1.1140499 | 0.00010738 | tripartite motif containing 22 |
| RUNX2 | 1.11381755 | 0.0038451 | RUNX family transcription factor 2 |
| LGALS2 | 1.11145654 | 0.00968657 | galectin 2 |
| LINC00839 | 1.10772883 | 0.00103138 | long intergenic non-protein coding RNA 839 |
| EREG | 1.09792808 | 8.44E-06 | epiregulin |
| RDH16 | 1.09787809 | 0.00255733 | retinol dehydrogenase 16 |
| GJA3 | 1.09502607 | 0.00215268 | gap junction protein alpha 3 |
| SKAP1 | 1.08999398 | 0.03074437 | src kinase associated phosphoprotein 1 |
| HMCN1 | 1.08916339 | 0.02949264 | hemicentin 1 |
| CPS1 | 1.08835305 | 1.50E-05 | carbamoyl-phosphate synthase 1 |
| FYB1 | 1.08138801 | 0.00154369 | FYN binding protein 1 |
| LYPD5 | 1.08098738 | 0.03764898 | LY6/PLAUR domain containing 5 |
| MYO1B | 1.07868351 | 1.27E-06 | myosin IB |
| FIGN | 1.07512584 | 0.00192006 | fidgetin, microtubule severing factor |
| TENT5C | 1.07508179 | 0.00124341 | terminal nucleotidyltransferase 5C |
| IL21R | 1.07498843 | 0.0205423 | interleukin 21 receptor |
| KLK1 | 1.07054563 | 0.00191904 | kallikrein 1 |
| ELOVL7 | 1.06659856 | 0.00301126 | ELOVL fatty acid elongase 7 |
| CTSC | 1.06418451 | 1.22E-08 | cathepsin C |
| C2orf16 | 1.06172737 | 0.0018636 | chromosome 2 open reading frame 16 |
| CFH | 1.06047467 | 0.02511612 | complement factor H |
| IRAG1 | 1.05709732 | 0.00010704 | inositol 1,4,5-triphosphate receptor associated 1 |
| PTPRZ1 | 1.05476272 | 6.45E-05 | protein tyrosine phosphatase receptor type Z1 |
| GBP5 | 1.05401351 | 0.00157671 | guanylate binding protein 5 |
| RASGRP1 | 1.05391107 | 0.00018816 | RAS guanyl releasing protein 1 |
| SULT1B1 | 1.04929644 | 0.02569773 | sulfotransferase family 1B member 1 |
| DYNAP | 1.04608185 | 0.02090427 | dynactin associated protein |
| GLUL | 1.03906272 | 0.00785909 | glutamate-ammonia ligase |
| MLF1 | 1.03830635 | 0.00060049 | myeloid leukemia factor 1 |
| GUCY1A1 | 1.03000666 | 0.02968902 | guanylate cyclase 1 soluble subunit alpha 1 |
| ZNF727 | 1.02919321 | 0.01839818 | zinc finger protein 727 |
| CLIC4 | 1.02632437 | 0.00343448 | chloride intracellular channel 4 |
| CLEC2D | 1.02235926 | 0.0061545 | C-type lectin domain family 2 member D |
| HLA-V | 1.01970459 | 0.01244413 | major histocompatibility complex, class I, V (pseudogene) |
| CA2 | 1.01731553 | 0.04285376 | carbonic anhydrase 2 |
| PDE4DIPP6 | 1.012055 | 0.03206596 | PDE4DIP pseudogene 6 |
| C1S | 1.01161269 | 0.02341762 | complement C1s |
| BCL2L15 | 1.01046272 | 0.01358281 | BCL2 like 15 |
| FFAR2 | 1.00710651 | 0.04004975 | free fatty acid receptor 2 |
| LAMP3 | 1.00703842 | 0.00354759 | lysosomal associated membrane protein 3 |
| PTHLH | 1.00686399 | 0.02850952 | parathyroid hormone like hormone |
| ETV7 | 0.99338452 | 0.00279323 | ETS variant transcription factor 7 |
| HDGFL3 | 0.98746544 | 0.0004811 | HDGF like 3 |
| CRACR2A | 0.98608052 | 0.04586702 | calcium release activated channel regulator 2A |
| TSHZ2 | 0.98442271 | 1.65E-06 | teashirt zinc finger homeobox 2 |
| CUZD1 | 0.9732655 | 0.02665424 | CUB and zona pellucida like domains 1 |
| DNAJB5 | 0.96879106 | 2.66E-07 | DnaJ heat shock protein family (Hsp40) member B5 |
| ITGAL | 0.96671216 | 0.02152672 | integrin subunit alpha L |
| HK2 | 0.96530148 | 0.00022202 | hexokinase 2 |
| MIR27B | 0.96453033 | 0.03527191 | microRNA 27b |
| SERPINB3 | 0.96301402 | 0.00133431 | serpin family B member 3 |
| CLEC7A | 0.95971639 | 0.00472322 | C-type lectin domain containing 7A |
| COL8A1 | 0.95965601 | 0.01454852 | collagen type VIII alpha 1 chain |
| ZNF286A | 0.95880824 | 1.29E-05 | zinc finger protein 286A |
| EHBP1-AS1 | 0.95073004 | 0.00430438 | EHBP1 antisense RNA 1 |
| NRP2 | 0.94971787 | 0.0011125 | neuropilin 2 |
| EDA2R | 0.94969019 | 0.02431152 | ectodysplasin A2 receptor |
| IGF2BP3 | 0.94184588 | 0.00133447 | insulin like growth factor 2 mRNA binding protein 3 |
| WNK3 | 0.94112189 | 0.01140983 | WNK lysine deficient protein kinase 3 |
| CDC42EP3 | 0.94047687 | 0.0110338 | CDC42 effector protein 3 |
| GCOM1 | 0.9391682 | 0.00158855 | GCOM1, MYZAP-POLR2M combined locus |
| SMAD9 | 0.93864034 | 0.04368755 | SMAD family member 9 |
| JAK3 | 0.9351081 | 0.00723014 | Janus kinase 3 |
| PDE7B | 0.93195533 | 0.01530916 | phosphodiesterase 7B |
| FZD4 | 0.92871906 | 0.00085133 | frizzled class receptor 4 |
| RASL12 | 0.92854661 | 0.00740385 | RAS like family 12 |
| FMO2 | 0.92554313 | 0.03635177 | flavin containing dimethylaniline monoxygenase 2 |
| TTC39B | 0.92482019 | 5.48E-05 | tetratricopeptide repeat domain 39B |
| TMEM178B | 0.92320375 | 0.01291067 | transmembrane protein 178B |
| PIK3R3 | 0.9226486 | 0.00161199 | phosphoinositide-3-kinase regulatory subunit 3 |
| SPAG4 | 0.91561099 | 0.03712287 | sperm associated antigen 4 |
| SIRPA | 0.90921633 | 0.00011753 | signal regulatory protein alpha |
| OAS2 | 0.90839899 | 0.02047635 | 2'-5'-oligoadenylate synthetase 2 |
| MIR4435-2HG | 0.90812133 | 0.00011015 | MIR4435-2 host gene |
| NPIPB4 | 0.90809235 | 0.04082376 | nuclear pore complex interacting protein family member B4 |
| RFTN2 | 0.9016301 | 0.03020502 | raftlin family member 2 |
| PWWP2B | -0.9004284 | 0.00153445 | PWWP domain containing 2B |
| GCNT1 | -0.9011143 | 0.00708139 | glucosaminyl (N-acetyl) transferase 1 |
| AIFM2 | -0.9051596 | 6.66E-05 | apoptosis inducing factor mitochondria associated 2 |
| DCST1 | -0.9063829 | 0.00629156 | DC-STAMP domain containing 1 |
| CAPN15 | -0.9065347 | 6.33E-13 | calpain 15 |
| MCRIP2 | -0.9081065 | 1.49E-10 | MAPK regulated corepressor interacting protein 2 |
| DHRS13 | -0.9087269 | 8.25E-06 | dehydrogenase/reductase 13 |
| OBSCN | -0.9098145 | 0.01460205 | obscurin, cytoskeletal calmodulin and titin-interacting RhoGEF |
| ZNF219 | -0.9101142 | 1.35E-12 | zinc finger protein 219 |
| THOC3 | -0.9116124 | 2.62E-07 | THO complex 3 |
| CHADL | -0.9138812 | 0.0025119 | chondroadherin like |
| MROH6 | -0.9156355 | 0.00274599 | maestro heat like repeat family member 6 |
| RILP | -0.9158818 | 1.01E-08 | Rab interacting lysosomal protein |
| ZNF321P | -0.9163259 | 0.02499533 | zinc finger protein 321, pseudogene |
| ZNF628 | -0.9177975 | 3.67E-07 | zinc finger protein 628 |
| RNF225 | -0.9179331 | 5.20E-05 | ring finger protein 225 |
| OSBP2 | -0.9185269 | 0.00414618 | oxysterol binding protein 2 |
| PODXL2 | -0.9187339 | 5.10E-09 | podocalyxin like 2 |
| SFTA2 | -0.9192253 | 0.0140231 | surfactant associated 2 |
| P2RY13 | -0.9203382 | 0.00725447 | purinergic receptor P2Y13 |
| ZDHHC1 | -0.9217334 | 4.02E-06 | zinc finger DHHC-type containing 1 |
| LAMA5 | -0.9218083 | 1.58E-11 | laminin subunit alpha 5 |
| VSIR | -0.9235446 | 1.59E-06 | V-set immunoregulatory receptor |
| MRPL41 | -0.9235618 | 1.69E-12 | mitochondrial ribosomal protein L41 |
| PRR22 | -0.9247374 | 0.00021416 | proline rich 22 |
| DUSP23 | -0.9254797 | 5.30E-14 | dual specificity phosphatase 23 |
| CCDC107 | -0.9257898 | 0.0103495 | coiled-coil domain containing 107 |
| COA6-AS1 | -0.9278011 | 0.0355505 | COA6 antisense RNA 1 |
| WASHC1 | -0.9284347 | 0.03551575 | WASH complex subunit 1 |
| DNPH1 | -0.9302308 | 8.37E-08 | 2'-deoxynucleoside 5'-phosphate N-hydrolase 1 |
| CCDC74A | -0.9339541 | 0.01016231 | coiled-coil domain containing 74A |
| YDJC | -0.9346408 | 7.04E-10 | YdjC chitooligosaccharide deacetylase homolog |
| MIB2 | -0.9390231 | 1.31E-14 | MIB E3 ubiquitin protein ligase 2 |
| CEBPD | -0.940396 | 1.85E-07 | CCAAT enhancer binding protein delta |
| GYPC | -0.9427947 | 0.0008647 | glycophorin C (Gerbich blood group) |
| ZNF865 | -0.9439801 | 8.29E-18 | zinc finger protein 865 |
| SMPD4BP | -0.9446212 | 2.22E-06 | sphingomyelin phosphodiesterase 4B, pseudogene |
| C1orf53 | -0.945351 | 0.01342808 | chromosome 1 open reading frame 53 |
| EML3 | -0.9459899 | 5.44E-32 | EMAP like 3 |
| MEX3D | -0.9473559 | 4.00E-12 | mex-3 RNA binding family member D |
| FGFBP3 | -0.9476002 | 0.00250221 | fibroblast growth factor binding protein 3 |
| FGF22 | -0.94946 | 0.02748529 | fibroblast growth factor 22 |
| AURKC | -0.9502267 | 0.03484213 | aurora kinase C |
| TMEM217 | -0.9507563 | 9.92E-06 | transmembrane protein 217 |
| FAM3D | -0.9540788 | 4.18E-12 | FAM3 metabolism regulating signaling molecule D |
| STUB1 | -0.955472 | 3.43E-19 | STIP1 homology and U-box containing protein 1 |
| MUC20 | -0.9609691 | 0.02780557 | mucin 20, cell surface associated |
| RNF126P1 | -0.9643274 | 0.01833629 | ring finger protein 126 pseudogene 1 |
| CFAP410 | -0.9667199 | 8.48E-07 | cilia and flagella associated protein 410 |
| INAFM1 | -0.9668218 | 4.92E-06 | InaF motif containing 1 |
| SCGB1A1 | -0.9677573 | 0.0012603 | secretoglobin family 1A member 1 |
| SDF2L1 | -0.9684636 | 1.04E-11 | stromal cell derived factor 2 like 1 |
| RGCC | -0.9713862 | 0.01555459 | regulator of cell cycle |
| H1-10 | -0.9722321 | 1.62E-11 | H1.10 linker histone |
| SLCO2B1 | -0.9766236 | 0.0067885 | solute carrier organic anion transporter family member 2B1 |
| TBKBP1 | -0.9837674 | 2.34E-14 | TBK1 binding protein 1 |
| ZNF414 | -0.9842447 | 1.74E-16 | zinc finger protein 414 |
| MTHFS | -0.9866445 | 0.03611912 | methenyltetrahydrofolate synthetase |
| ARHGAP33 | -0.9870173 | 3.22E-06 | Rho GTPase activating protein 33 |
| GMDS | -0.9880254 | 0.00058145 | GDP-mannose 4,6-dehydratase |
| IGFBP2 | -0.991044 | 2.16E-10 | insulin like growth factor binding protein 2 |
| FALEC | -0.9912413 | 0.00416498 | focally amplified long non-coding RNA in epithelial cancer |
| DBNDD2 | -0.9917699 | 0.00238201 | dysbindin domain containing 2 |
| FMO9P | -0.9922016 | 0.03005585 | flavin containing dimethylaniline monoxygenase 9, pseudogene |
| NRG4 | -0.9922383 | 0.02019333 | neuregulin 4 |
| PRRT4 | -0.9963844 | 0.03138708 | proline rich transmembrane protein 4 |
| MFSD3 | -1.0013693 | 5.64E-12 | major facilitator superfamily domain containing 3 |
| LRFN2 | -1.0073206 | 0.00043492 | leucine rich repeat and fibronectin type III domain containing 2 |
| LINC00857 | -1.0081573 | 0.01697578 | long intergenic non-protein coding RNA 857 |
| ALKBH7 | -1.0116331 | 6.80E-20 | alkB homolog 7 |
| S100A6 | -1.013719 | 1.50E-05 | S100 calcium binding protein A6 |
| PDLIM4 | -1.0146345 | 2.61E-06 | PDZ and LIM domain 4 |
| NOXA1 | -1.0164234 | 2.85E-08 | NADPH oxidase activator 1 |
| MSR1 | -1.0169748 | 0.04441245 | macrophage scavenger receptor 1 |
| FCGR2B | -1.0170623 | 0.00022033 | Fc fragment of IgG receptor IIb |
| CYP2F1 | -1.0185213 | 0.00021543 | cytochrome P450 family 2 subfamily F member 1 |
| MAPK8IP1 | -1.0210974 | 0.01795605 | mitogen-activated protein kinase 8 interacting protein 1 |
| MYADM | -1.0219262 | 0.0003609 | myeloid associated differentiation marker |
| FBXL15 | -1.0222239 | 2.19E-10 | F-box and leucine rich repeat protein 15 |
| PDE1A | -1.023666 | 0.00616546 | phosphodiesterase 1A |
| LGALS3 | -1.0303285 | 2.14E-06 | galectin 3 |
| CCDC85B | -1.0304909 | 2.87E-08 | coiled-coil domain containing 85B |
| NKD2 | -1.0305168 | 0.04422016 | NKD inhibitor of WNT signaling pathway 2 |
| PPDPF | -1.030547 | 4.02E-07 | pancreatic progenitor cell differentiation and proliferation factor |
| PRSS36 | -1.0326516 | 0.00021636 | serine protease 36 |
| FADD | -1.0370472 | 2.90E-09 | Fas associated via death domain |
| HPDL | -1.0381082 | 0.00013098 | 4-hydroxyphenylpyruvate dioxygenase like |
| PLD4 | -1.039773 | 0.00927066 | phospholipase D family member 4 |
| COMTD1 | -1.0414334 | 1.75E-26 | catechol-O-methyltransferase domain containing 1 |
| PRDX6-AS1 | -1.0430327 | 0.00323191 | PRDX6 antisense RNA 1 |
| DOCK8-AS1 | -1.0491132 | 0.02602365 | DOCK8 antisense RNA 1 |
| EPB41L4A-DT | -1.056264 | 0.00010808 | EPB41L4A divergent transcript |
| ALCAM | -1.0569008 | 0.00013841 | activated leukocyte cell adhesion molecule |
| FAM107A | -1.0576977 | 0.0433508 | family with sequence similarity 107 member A |
| TJP2 | -1.0578808 | 9.14E-11 | tight junction protein 2 |
| HSD17B1-AS1 | -1.0581359 | 0.00447831 | HSD17B1 antisense RNA 1 |
| RTN1 | -1.058253 | 0.00335329 | reticulon 1 |
| LINC01140 | -1.0670745 | 0.02399561 | long intergenic non-protein coding RNA 1140 |
| ADAM11 | -1.0686278 | 0.00112887 | ADAM metallopeptidase domain 11 |
| LOC100129534 | -1.0686325 | 0.01354354 | small nuclear ribonucleoprotein polypeptide N pseudogene |
| MAN1B1-DT | -1.0750183 | 0.00770448 | MAN1B1 divergent transcript |
| REX1BD | -1.0750522 | 2.35E-22 | required for excision 1-B domain containing |
| CLEC11A | -1.0756074 | 2.97E-05 | C-type lectin domain containing 11A |
| CRACR2B | -1.0764108 | 0.0004122 | calcium release activated channel regulator 2B |
| CNTNAP3 | -1.0766858 | 0.00321495 | contactin associated protein family member 3 |
| NUDT8 | -1.0787743 | 8.29E-18 | nudix hydrolase 8 |
| LINC01023 | -1.0806409 | 3.95E-05 | long intergenic non-protein coding RNA 1023 |
| PRR7 | -1.0844419 | 6.28E-05 | proline rich 7, synaptic |
| UBALD1 | -1.0874566 | 2.97E-09 | UBA like domain containing 1 |
| PORCN | -1.0883441 | 0.01700474 | porcupine O-acyltransferase |
| ZNF837 | -1.0884215 | 2.61E-07 | zinc finger protein 837 |
| SLC34A3 | -1.1038621 | 0.00702053 | solute carrier family 34 member 3 |
| CAVIN3 | -1.1056625 | 5.47E-05 | caveolae associated protein 3 |
| VSIG2 | -1.1084584 | 8.62E-09 | V-set and immunoglobulin domain containing 2 |
| ZNF358 | -1.1090553 | 1.44E-08 | zinc finger protein 358 |
| TXNDC5 | -1.1091948 | 0.005471 | thioredoxin domain containing 5 |
| RCOR2 | -1.1146992 | 0.01494686 | REST corepressor 2 |
| METRN | -1.116747 | 2.21E-07 | meteorin, glial cell differentiation regulator |
| SYNGR3 | -1.1188918 | 0.02803448 | synaptogyrin 3 |
| RHOF | -1.1232925 | 0.01376723 | ras homolog family member F, filopodia associated |
| CLDN23 | -1.1261829 | 1.14E-05 | claudin 23 |
| LINC01003 | -1.1289752 | 7.81E-08 | long intergenic non-protein coding RNA 1003 |
| TMEM63C | -1.1316117 | 1.16E-05 | transmembrane protein 63C |
| SMIM22 | -1.1479497 | 0.01799557 | small integral membrane protein 22 |
| CTU1 | -1.1491212 | 9.94E-09 | cytosolic thiouridylase subunit 1 |
| EPIST | -1.1494229 | 4.58E-12 | esophagus epithelial intergenic associated transcript |
| C16orf74 | -1.1517468 | 0.00332511 | chromosome 16 open reading frame 74 |
| MXRA8 | -1.1539902 | 5.25E-08 | matrix remodeling associated 8 |
| ATAD3C | -1.1631971 | 0.03400633 | ATPase family AAA domain containing 3C |
| ADAP1 | -1.166979 | 0.00478283 | ArfGAP with dual PH domains 1 |
| OBSCN-AS1 | -1.1689939 | 0.01429771 | OBSCN antisense RNA 1 |
| SIX5 | -1.1693077 | 2.31E-10 | SIX homeobox 5 |
| LINC00863 | -1.1710189 | 0.00261226 | long intergenic non-protein coding RNA 863 |
| CDH20 | -1.1737525 | 0.03039959 | cadherin 20 |
| SCX | -1.1743609 | 0.0011991 | scleraxis bHLH transcription factor |
| NEFH | -1.1808406 | 0.02051571 | neurofilament heavy chain |
| KIFC2 | -1.1827319 | 6.34E-07 | kinesin family member C2 |
| CEL | -1.1831955 | 1.08E-05 | carboxyl ester lipase |
| RNF224 | -1.1886762 | 0.00012043 | ring finger protein 224 |
| METTL27 | -1.1924519 | 0.03120616 | methyltransferase like 27 |
| MUC5B | -1.1951846 | 0.01866784 | mucin 5B, oligomeric mucus/gel-forming |
| CX3CR1 | -1.2027102 | 0.00642404 | C-X3-C motif chemokine receptor 1 |
| AATK | -1.2047885 | 0.00013436 | apoptosis associated tyrosine kinase |
| LOC100129484 | -1.2145463 | 0.00168198 | uncharacterized LOC100129484 |
| UBE2S | -1.2175986 | 7.57E-07 | ubiquitin conjugating enzyme E2 S |
| FJX1 | -1.2210276 | 5.31E-09 | four-jointed box kinase 1 |
| JUND | -1.2286382 | 1.36E-12 | JunD proto-oncogene, AP-1 transcription factor subunit |
| SNX31 | -1.233601 | 0.00983859 | sorting nexin 31 |
| TMEM200B | -1.2373374 | 0.00131962 | transmembrane protein 200B |
| MGAT2 | -1.2380516 | 1.76E-05 | alpha-1,6-mannosyl-glycoprotein 2-beta-N-acetylglucosaminyltransferase |
| PPP1R16A | -1.2422872 | 5.68E-16 | protein phosphatase 1 regulatory subunit 16A |
| SCAND1 | -1.2430469 | 6.43E-49 | SCAN domain containing 1 |
| SPATC1L | -1.2466171 | 0.00534851 | spermatogenesis and centriole associated 1 like |
| GLTPD2 | -1.2531821 | 6.88E-05 | glycolipid transfer protein domain containing 2 |
| SLC35F3 | -1.2593266 | 0.00651236 | solute carrier family 35 member F3 |
| DUOXA2 | -1.2648101 | 0.00756716 | dual oxidase maturation factor 2 |
| ADAT3 | -1.2652608 | 3.21E-09 | adenosine deaminase tRNA specific 3 |
| ARL2BP | -1.2668807 | 8.87E-06 | ADP ribosylation factor like GTPase 2 binding protein |
| TSPAN12 | -1.2728025 | 6.51E-07 | tetraspanin 12 |
| NR1D1 | -1.2835866 | 0.01254225 | nuclear receptor subfamily 1 group D member 1 |
| KCP | -1.2861675 | 2.12E-07 | kielin cysteine rich BMP regulator |
| ACOT4 | -1.2891288 | 0.00025565 | acyl-CoA thioesterase 4 |
| BCAS1 | -1.2899345 | 0.00018674 | brain enriched myelin associated protein 1 |
| ATP13A2 | -1.3024205 | 0.00066157 | ATPase cation transporting 13A2 |
| LNC-LBCS | -1.3041219 | 0.00123609 | lncRNA bladder and prostate cancer suppressor, hnRNPK interacting |
| AMN | -1.3086567 | 3.74E-18 | amnion associated transmembrane protein |
| KLF2 | -1.3154545 | 0.00016598 | Kruppel like factor 2 |
| CYP4F35P | -1.3188395 | 0.02127578 | cytochrome P450 family 4 subfamily F member 35, pseudogene |
| ADAD2 | -1.3200402 | 2.00E-10 | adenosine deaminase domain containing 2 |
| PSCA | -1.3223589 | 0.01460205 | prostate stem cell antigen |
| ANTKMT | -1.3534964 | 1.80E-08 | adenine nucleotide translocase lysine methyltransferase |
| SLC49A3 | -1.3597868 | 4.04E-05 | solute carrier family 49 member 3 |
| FAM171A2 | -1.3638642 | 1.32E-05 | family with sequence similarity 171 member A2 |
| TOB1-AS1 | -1.3783912 | 0.00191981 | TOB1 antisense RNA 1 |
| CST6 | -1.3887375 | 2.62E-07 | cystatin E/M |
| NKX2-8 | -1.4032037 | 2.62E-06 | NK2 homeobox 8 |
| NUDT3 | -1.4182893 | 2.52E-07 | nudix hydrolase 3 |
| HES4 | -1.425267 | 1.06E-08 | hes family bHLH transcription factor 4 |
| KCNK13 | -1.447499 | 0.0084576 | potassium two pore domain channel subfamily K member 13 |
| GET4 | -1.4627591 | 8.85E-10 | guided entry of tail-anchored proteins factor 4 |
| CRIP2 | -1.4667074 | 1.80E-10 | cysteine rich protein 2 |
| LOC100130449 | -1.4719648 | 2.44E-11 | uncharacterized LOC100130449 |
| DMRTA2 | -1.4895726 | 0.01231476 | DMRT like family A2 |
| UFSP1 | -1.4956288 | 0.00020374 | UFM1 specific peptidase 1 (inactive) |
| FZD8 | -1.507085 | 2.58E-07 | frizzled class receptor 8 |
| CLEC18B | -1.5110179 | 0.01812126 | C-type lectin domain family 18 member B |
| LINC01587 | -1.5156 | 0.00105153 | long intergenic non-protein coding RNA 1587 |
| PLAC8 | -1.5214245 | 0.00621779 | placenta associated 8 |
| TMEM238 | -1.5381304 | 3.34E-21 | transmembrane protein 238 |
| METTL24 | -1.5426228 | 0.00138818 | methyltransferase like 24 |
| RNF103-CHMP3 | -1.5578798 | 0.00024673 | RNF103-CHMP3 readthrough |
| PART1 | -1.5611961 | 3.63E-05 | prostate androgen-regulated transcript 1 |
| ETNK2 | -1.5626683 | 0.00158168 | ethanolamine kinase 2 |
| CITED4 | -1.5733939 | 2.58E-11 | Cbp/p300 interacting transactivator with Glu/Asp rich carboxy-terminal domain 4 |
| CRYM | -1.576304 | 0.00208344 | crystallin mu |
| PDE6A | -1.5988994 | 3.95E-05 | phosphodiesterase 6A |
| SPON2 | -1.6095942 | 0.00013885 | spondin 2 |
| FAM181B | -1.6145426 | 0.00015571 | family with sequence similarity 181 member B |
| ZFPM1 | -1.6220184 | 3.76E-23 | zinc finger protein, FOG family member 1 |
| CSF3R | -1.6320112 | 0.00959628 | colony stimulating factor 3 receptor |
| LOC729966 | -1.6389928 | 0.03274667 | uncharacterized LOC729966 |
| GADD45G | -1.6581841 | 1.05E-06 | growth arrest and DNA damage inducible gamma |
| LINC02471 | -1.6644589 | 0.00989251 | long intergenic non-protein coding RNA 2471 |
| GNA14 | -1.677462 | 6.97E-07 | G protein subunit alpha 14 |
| OXGR1 | -1.695641 | 0.00103404 | oxoglutarate receptor 1 |
| CLEC4F | -1.6963131 | 1.01E-05 | C-type lectin domain family 4 member F |
| BASP1 | -1.6974832 | 2.35E-16 | brain abundant membrane attached signal protein 1 |
| CREB5 | -1.6996912 | 0.00035817 | cAMP responsive element binding protein 5 |
| POMC | -1.715093 | 0.00047954 | proopiomelanocortin |
| HERC2P3 | -1.7623888 | 0.02274464 | HERC2 pseudogene 3 |
| TNNI2 | -1.762672 | 0.00666186 | troponin I2, fast skeletal type |
| DKK1 | -1.7685317 | 0.02188923 | dickkopf WNT signaling pathway inhibitor 1 |
| IRAG2 | -1.7695082 | 0.0008665 | inositol 1,4,5-triphosphate receptor associated 2 |
| ARHGAP39 | -1.7724716 | 1.71E-09 | Rho GTPase activating protein 39 |
| JUN | -1.7733036 | 2.87E-10 | Jun proto-oncogene, AP-1 transcription factor subunit |
| TPGS1 | -1.7784604 | 7.96E-13 | tubulin polyglutamylase complex subunit 1 |
| GEM | -1.7978988 | 0.04084478 | GTP binding protein overexpressed in skeletal muscle |
| IGFBP6 | -1.8364429 | 0.00294872 | insulin like growth factor binding protein 6 |
| FBP1 | -1.8504547 | 0.02048337 | fructose-bisphosphatase 1 |
| MAMDC2 | -1.8831536 | 6.50E-05 | MAM domain containing 2 |
| WNT7A | -1.8978513 | 0.00122572 | Wnt family member 7A |
| ENDOG | -1.8994446 | 1.73E-10 | endonuclease G |
| SPSB3 | -1.9131746 | 4.88E-07 | splA/ryanodine receptor domain and SOCS box containing 3 |
| NR4A1 | -1.9224974 | 0.04212415 | nuclear receptor subfamily 4 group A member 1 |
| MC5R | -1.946219 | 0.00061557 | melanocortin 5 receptor |
| LAMB4 | -1.9484512 | 1.30E-06 | laminin subunit beta 4 |
| LINC02099 | -1.9493456 | 0.00851323 | long intergenic non-protein coding RNA 2099 |
| SYT8 | -1.9786255 | 0.00018674 | synaptotagmin 8 |
| TMEM160 | -1.9922355 | 8.60E-34 | transmembrane protein 160 |
| PDF | -2.007821 | 9.64E-09 | peptide deformylase, mitochondrial |
| FTH1 | -2.0653616 | 1.77E-11 | ferritin heavy chain 1 |
| RAET1E-AS1 | -2.0777489 | 1.95E-07 | RAET1E antisense RNA 1 |
| SRXN1 | -2.0829618 | 9.22E-06 | sulfiredoxin 1 |
| RAB5IF | -2.1331798 | 2.04E-34 | RAB5 interacting factor |
| LYPD2 | -2.1357265 | 4.44E-17 | LY6/PLAUR domain containing 2 |
| PEG3 | -2.1622284 | 0.00030673 | paternally expressed 3 |
| CRIP1 | -2.2090391 | 1.84E-05 | cysteine rich protein 1 |
| PRR15 | -2.2094314 | 0.00231593 | proline rich 15 |
| HBA2 | -2.2263561 | 7.91E-08 | hemoglobin subunit alpha 2 |
| LRRC14 | -2.2331185 | 1.21E-07 | leucine rich repeat containing 14 |
| MESP1 | -2.26886 | 2.45E-06 | mesoderm posterior bHLH transcription factor 1 |
| CRTAC1 | -2.3361791 | 4.35E-08 | cartilage acidic protein 1 |
| FAM20A | -2.3895494 | 0.00400099 | FAM20A golgi associated secretory pathway pseudokinase |
| TNNT3 | -2.429342 | 0.00012227 | troponin T3, fast skeletal type |
| NPPC | -2.4554908 | 0.00063803 | natriuretic peptide C |
| GPT | -2.4785574 | 1.60E-10 | glutamic--pyruvic transaminase |
| C8orf82 | -2.5501001 | 5.75E-15 | chromosome 8 open reading frame 82 |
| PTGS2 | -2.5627747 | 0.00968749 | prostaglandin-endoperoxide synthase 2 |
| EDN3 | -2.7122808 | 2.09E-07 | endothelin 3 |
| MIR149 | -2.8501206 | 1.02E-07 | microRNA 149 |
| AQP5 | -2.9153898 | 0.01183698 | aquaporin 5 |
| MUC22 | -2.9857864 | 0.00023688 | mucin 22 |
| EGR2 | -3.1750887 | 0.01676044 | early growth response 2 |
| RECQL4 | -3.2417918 | 7.04E-10 | RecQ like helicase 4 |
| NOG | -3.2520618 | 1.21E-07 | noggin |
| SLITRK5 | -3.294499 | 1.33E-06 | SLIT and NTRK like family member 5 |
| EGR1 | -3.2958648 | 9.93E-07 | early growth response 1 |
| TPPP3 | -3.3939972 | 7.29E-06 | tubulin polymerization promoting protein family member 3 |
| FOSB | -4.6177132 | 2.97E-05 | FosB proto-oncogene, AP-1 transcription factor subunit |
| FOS | -4.6644715 | 5.27E-09 | Fos proto-oncogene, AP-1 transcription factor subunit |
| DMRT3 | -4.7621944 | 0.00015014 | doublesex and mab-3 related transcription factor 3 |

**Supplementary Table 4. Genes differentially expressed in distal esophagus of type 2 achalasia vs. healthy control**

| **Gene Symbol** | **Log2 Fold Change** | **FDR Adj p Value** | **Description** |
| --- | --- | --- | --- |
| MMP12 | 3.52432701 | 0.00072109 | matrix metallopeptidase 12 |
| NEFM | 3.51325692 | 0.02951065 | neurofilament medium chain |
| APOBEC3A | 3.24855281 | 0.00092633 | apolipoprotein B mRNA editing enzyme catalytic subunit 3A |
| APOBEC3A_B | 3.24855281 | 0.00092633 | APOBEC3A and APOBEC3B deletion hybrid |
| KRT1 | 3.23757918 | 0.01986909 | keratin 1 |
| GFPT2 | 3.07518688 | 0.00042816 | glutamine-fructose-6-phosphate transaminase 2 |
| NEFL | 2.95344998 | 0.03621295 | neurofilament light chain |
| HMGA2 | 2.84768049 | 0.00163489 | high mobility group AT-hook 2 |
| LIPG | 2.79531383 | 0.00545542 | lipase G, endothelial type |
| ADGRL3 | 2.4820856 | 0.03603125 | adhesion G protein-coupled receptor L3 |
| APBA2 | 2.4672165 | 0.00917271 | amyloid beta precursor protein binding family A member 2 |
| TMEM35A | 2.33020417 | 0.00703888 | transmembrane protein 35A |
| GLDC | 2.14409698 | 0.0122022 | glycine decarboxylase |
| LIPK | 1.98082067 | 0.00386993 | lipase family member K |
| PLA2G4E | 1.9637568 | 0.00152852 | phospholipase A2 group IVE |
| CARD18 | 1.96204748 | 0.00120225 | caspase recruitment domain family member 18 |
| NRCAM | 1.91038237 | 0.0154898 | neuronal cell adhesion molecule |
| CFAP251 | 1.89957813 | 0.0069772 | cilia and flagella associated protein 251 |
| PHLDB2 | 1.86949984 | 0.00038937 | pleckstrin homology like domain family B member 2 |
| PTPRD | 1.86157569 | 0.0004968 | protein tyrosine phosphatase receptor type D |
| DDIT4 | 1.86069042 | 0.00072141 | DNA damage inducible transcript 4 |
| MIR31HG | 1.84162674 | 0.01618568 | MIR31 host gene |
| SEMA3C | 1.78735338 | 0.02910998 | semaphorin 3C |
| THBS2 | 1.7663029 | 0.04568302 | thrombospondin 2 |
| PNLIPRP3 | 1.76095277 | 0.01319817 | pancreatic lipase related protein 3 |
| RASL12 | 1.7604794 | 0.02205095 | RAS like family 12 |
| CEMIP | 1.75876149 | 0.02131332 | cell migration inducing hyaluronidase 1 |
| HAL | 1.75035472 | 0.0048501 | histidine ammonia-lyase |
| COL4A6 | 1.68512294 | 0.02823735 | collagen type IV alpha 6 chain |
| CAPN6 | 1.61192766 | 0.00053764 | calpain 6 |
| DNAJB5 | 1.57419056 | 0.00027728 | DnaJ heat shock protein family (Hsp40) member B5 |
| SERPINE1 | 1.54488373 | 0.00929502 | serpin family E member 1 |
| IL33 | 1.5001984 | 0.01050485 | interleukin 33 |
| DZIP1 | 1.48677641 | 0.00022576 | DAZ interacting zinc finger protein 1 |
| STRA6 | 1.48002487 | 0.04265586 | signaling receptor and transporter of retinol STRA6 |
| CDH17 | 1.40907711 | 0.04265586 | cadherin 17 |
| CDH16 | 1.40732334 | 0.00512155 | cadherin 16 |
| SLC15A1 | 1.38064285 | 0.0086121 | solute carrier family 15 member 1 |
| RDH16 | 1.36917763 | 0.04054882 | retinol dehydrogenase 16 |
| RGS20 | 1.36781988 | 0.00739793 | regulator of G protein signaling 20 |
| PTHLH | 1.35705147 | 0.04202966 | parathyroid hormone like hormone |
| PGBD5 | 1.32662213 | 0.02624518 | piggyBac transposable element derived 5 |
| SORCS2 | 1.31888832 | 0.00032492 | sortilin related VPS10 domain containing receptor 2 |
| PRRX1 | 1.31439825 | 0.04630598 | paired related homeobox 1 |
| MYO1B | 1.3134395 | 0.00154261 | myosin IB |
| WNT10B | 1.27444103 | 0.04630598 | Wnt family member 10B |
| C1orf216 | 1.25417552 | 0.00750444 | chromosome 1 open reading frame 216 |
| CIB2 | 1.24183434 | 0.00904472 | calcium and integrin binding family member 2 |
| ATP13A5 | 1.24024805 | 0.02624518 | ATPase 13A5 |
| NRP2 | 1.22840464 | 0.02152746 | neuropilin 2 |
| MARCO | 1.22213308 | 0.00162046 | macrophage receptor with collagenous structure |
| GGT1 | 1.19823656 | 0.0197453 | gamma-glutamyltransferase 1 |
| KLK1 | 1.19117446 | 0.0197453 | kallikrein 1 |
| ADTRP | 1.18783361 | 0.00775605 | androgen dependent TFPI regulating protein |
| CPA3 | 1.17339682 | 0.04604626 | carboxypeptidase A3 |
| KRT16 | 1.16686704 | 0.03555703 | keratin 16 |
| ARNT2 | 1.13977433 | 0.00303605 | aryl hydrocarbon receptor nuclear translocator 2 |
| SDK2 | 1.13516912 | 0.04604626 | sidekick cell adhesion molecule 2 |
| EPOP | 1.09197001 | 0.02305231 | elongin BC and polycomb repressive complex 2 associated protein |
| GSDME | 1.08794994 | 0.02492939 | gasdermin E |
| PKIG | 1.08655985 | 0.0340249 | cAMP-dependent protein kinase inhibitor gamma |
| SYT7 | 1.07100095 | 0.02068246 | synaptotagmin 7 |
| EPPK1 | 1.04386998 | 0.02624518 | epiplakin 1 |
| LRRC8E | 1.03636161 | 0.03419207 | leucine rich repeat containing 8 VRAC subunit E |
| PARVB | 1.02905413 | 0.0342445 | parvin beta |
| KRT6C | 1.02289066 | 0.04498848 | keratin 6C |
| SERPINB9 | 1.00978872 | 0.00098574 | serpin family B member 9 |
| EREG | 1.00091926 | 0.02910998 | epiregulin |
| TMEM38A | 0.99414835 | 0.00750444 | transmembrane protein 38A |
| LINC00933 | 0.9775807 | 0.04108329 | long intergenic non-protein coding RNA 933 |
| HK2 | 0.97547021 | 0.03801563 | hexokinase 2 |
| FIGN | 0.95411789 | 0.04996758 | fidgetin, microtubule severing factor |
| STARD4 | 0.93524264 | 0.02900973 | StAR related lipid transfer domain containing 4 |
| PFN2 | 0.91622927 | 0.00794529 | profilin 2 |
| HUNK | 0.90671566 | 0.04054882 | hormonally up-regulated Neu-associated kinase |
| PTPRZ1 | 0.90381661 | 0.02988088 | protein tyrosine phosphatase receptor type Z1 |
| ENAH | 0.90023886 | 0.00071112 | ENAH actin regulator |
| CLDN23 | -0.9000951 | 0.00208633 | claudin 23 |
| ANKS1B | -0.9143921 | 0.04250772 | ankyrin repeat and sterile alpha motif domain containing 1B |
| FGFBP3 | -0.9240786 | 0.00735791 | fibroblast growth factor binding protein 3 |
| SLC6A4 | -0.9358451 | 0.00386993 | solute carrier family 6 member 4 |
| LOC105371267 | -0.9443837 | 0.03298181 | p53-regulated lncRNA 1 |
| RND3 | -0.9569078 | 0.01070801 | Rho family GTPase 3 |
| CTNNAL1 | -0.9571859 | 0.01050485 | catenin alpha like 1 |
| LRFN2 | -0.9610682 | 0.00071112 | leucine rich repeat and fibronectin type III domain containing 2 |
| CSF1R | -0.968323 | 4.31E-11 | colony stimulating factor 1 receptor |
| CYP2F1 | -0.9704349 | 0.00703888 | cytochrome P450 family 2 subfamily F member 1 |
| SNTB1 | -0.9859715 | 0.02022786 | syntrophin beta 1 |
| ID4 | -0.994849 | 0.00061515 | inhibitor of DNA binding 4, HLH protein |
| FCGR2A | -0.9998571 | 0.02152746 | Fc fragment of IgG receptor IIa |
| TPPP | -1.0323794 | 0.0197453 | tubulin polymerization promoting protein |
| F3 | -1.0501457 | 0.02239454 | coagulation factor III, tissue factor |
| DEPTOR | -1.0742635 | 0.00041234 | DEP domain containing MTOR interacting protein |
| PLAC4 | -1.0760777 | 0.0479255 | placenta enriched 4 |
| ARHGAP39 | -1.0823439 | 0.00194745 | Rho GTPase activating protein 39 |
| GNA14 | -1.0904369 | 0.03226522 | G protein subunit alpha 14 |
| LY6K | -1.0935346 | 0.02230625 | lymphocyte antigen 6 family member K |
| LOC102724163 | -1.1150812 | 0.0340249 | uncharacterized LOC102724163 |
| FFAR4 | -1.1218621 | 0.00945327 | free fatty acid receptor 4 |
| BASP1 | -1.1709138 | 0.00225804 | brain abundant membrane attached signal protein 1 |
| KCNMA1 | -1.1829753 | 0.03002482 | potassium calcium-activated channel subfamily M alpha 1 |
| LRRC7 | -1.1906481 | 0.03254321 | leucine rich repeat containing 7 |
| FCGR2B | -1.1912346 | 2.59E-05 | Fc fragment of IgG receptor IIb |
| JUN | -1.1940459 | 0.00053343 | Jun proto-oncogene, AP-1 transcription factor subunit |
| RTN1 | -1.2051632 | 0.00074394 | reticulon 1 |
| MNDA | -1.2321808 | 0.00163489 | myeloid cell nuclear differentiation antigen |
| P2RY13 | -1.2728993 | 0.0006125 | purinergic receptor P2Y13 |
| SIDT1 | -1.3127686 | 0.00027728 | SID1 transmembrane family member 1 |
| LYPD2 | -1.32235 | 0.00246684 | LY6/PLAUR domain containing 2 |
| ENPP2 | -1.335231 | 0.01214771 | ectonucleotide pyrophosphatase/phosphodiesterase 2 |
| PSCA | -1.3506089 | 0.0340249 | prostate stem cell antigen |
| CREB5 | -1.362505 | 0.04265586 | cAMP responsive element binding protein 5 |
| LRRC14 | -1.376396 | 0.03005961 | leucine rich repeat containing 14 |
| CRYM | -1.382877 | 0.0245917 | crystallin mu |
| LINC01587 | -1.4396354 | 0.00877246 | long intergenic non-protein coding RNA 1587 |
| CLEC4F | -1.4450038 | 0.00138627 | C-type lectin domain family 4 member F |
| C8orf82 | -1.4576335 | 0.01223828 | chromosome 8 open reading frame 82 |
| HBA2 | -1.4650292 | 0.01795236 | hemoglobin subunit alpha 2 |
| LNC-LBCS | -1.478256 | 0.00190092 | lncRNA bladder and prostate cancer suppressor, hnRNPK interacting |
| CX3CR1 | -1.5443728 | 0.00074394 | C-X3-C motif chemokine receptor 1 |
| TNNT3 | -1.7106097 | 0.01319817 | troponin T3, fast skeletal type |
| FMO9P | -1.7112088 | 0.0020338 | flavin containing dimethylaniline monoxygenase 9, pseudogene |
| CACNA1G | -1.7362465 | 0.00739793 | calcium voltage-gated channel subunit alpha1 G |
| REEP1 | -1.7392461 | 0.00053764 | receptor accessory protein 1 |
| CRTAC1 | -1.7460528 | 0.01607967 | cartilage acidic protein 1 |
| CRIP1 | -1.7760551 | 0.00361702 | cysteine rich protein 1 |
| RECQL4 | -1.8575281 | 0.04265586 | RecQ like helicase 4 |
| PDLIM3 | -1.9282545 | 0.02953484 | PDZ and LIM domain 3 |
| NR4A1 | -2.0007737 | 0.00795802 | nuclear receptor subfamily 4 group A member 1 |
| DPYSL3 | -2.0109556 | 0.02032512 | dihydropyrimidinase like 3 |
| LAMB4 | -2.016212 | 0.00945327 | laminin subunit beta 4 |
| MAMDC2 | -2.1153834 | 0.00029515 | MAM domain containing 2 |
| CYP2W1 | -2.1204747 | 0.02022786 | cytochrome P450 family 2 subfamily W member 1 |
| ATF3 | -2.1799558 | 1.47E-05 | activating transcription factor 3 |
| OXGR1 | -2.2421434 | 0.00053764 | oxoglutarate receptor 1 |
| IRAG2 | -2.3356194 | 7.24E-07 | inositol 1,4,5-triphosphate receptor associated 2 |
| SLITRK5 | -2.5499983 | 0.00236164 | SLIT and NTRK like family member 5 |
| MUC22 | -2.7494955 | 0.00361702 | mucin 22 |
| PTGS2 | -2.8742448 | 0.00965052 | prostaglandin-endoperoxide synthase 2 |
| TPPP3 | -3.0513126 | 2.08E-05 | tubulin polymerization promoting protein family member 3 |
| FOS | -3.181281 | 0.00330713 | Fos proto-oncogene, AP-1 transcription factor subunit |
| EGR2 | -3.1892467 | 0.03379558 | early growth response 2 |
| AQP5 | -3.560583 | 0.00136012 | aquaporin 5 |
| FOSB | -4.2261504 | 5.86E-05 | FosB proto-oncogene, AP-1 transcription factor subunit |

**Supplementary Table 5. Genes differentially expressed in proximal esophagus of type 1 achalasia vs. healthy control**

| **Gene Symbol** | **Log2 Fold Change** | **FDR Adj p Value** | **Description** |
| --- | --- | --- | --- |
| LCE3A | 5.89695964 | 0.00031001 | late cornified envelope 3A |
| PALM | 4.18154143 | 0.00069546 | paralemmin |
| SPRR2G | 3.73046441 | 0.00702287 | small proline rich protein 2G |
| LINC02028 | 3.44163588 | 0.00027279 | long intergenic non-protein coding RNA 2028 |
| MISP | 3.18421423 | 0.01911522 | mitotic spindle positioning |
| LINC02026 | 3.09422162 | 0.00248271 | long intergenic non-protein coding RNA 2026 |
| LCE3E | 3.07787765 | 0.0378553 | late cornified envelope 3E |
| WFDC12 | 3.06754499 | 0.01340309 | WAP four-disulfide core domain 12 |
| GLDC | 2.82701904 | 0.01715211 | glycine decarboxylase |
| STRA6 | 2.8253111 | 0.005678 | signaling receptor and transporter of retinol STRA6 |
| MARCO | 2.80301757 | 0.00149721 | macrophage receptor with collagenous structure |
| ARC | 2.70725967 | 0.01340309 | activity regulated cytoskeleton associated protein |
| MMP12 | 2.61355336 | 0.03553373 | matrix metallopeptidase 12 |
| LINC02188 | 2.45342615 | 0.00553018 | long intergenic non-protein coding RNA 2188 |
| NYAP1 | 2.39173743 | 0.01033807 | neuronal tyrosine phosphorylated phosphoinositide-3-kinase adaptor 1 |
| TPSD1 | 2.39033599 | 0.00167064 | tryptase delta 1 |
| FAM180A | 2.38521773 | 0.01519385 | family with sequence similarity 180 member A |
| KRTDAP | 2.33985079 | 0.03811099 | keratinocyte differentiation associated protein |
| KLHDC7B | 2.30709538 | 0.00333339 | kelch domain containing 7B |
| PLA2G4E | 2.26765973 | 0.01681754 | phospholipase A2 group IVE |
| PGBD5 | 2.23180315 | 6.20E-07 | piggyBac transposable element derived 5 |
| PI3 | 2.22078326 | 0.02205755 | peptidase inhibitor 3 |
| TMEM45A | 2.20673073 | 0.0002209 | transmembrane protein 45A |
| CARD17 | 2.18338494 | 0.00077976 | caspase recruitment domain family member 17 |
| MMP9 | 2.09535909 | 0.00473531 | matrix metallopeptidase 9 |
| RN7SL2 | 2.09227576 | 1.93E-05 | RNA component of signal recognition particle 7SL2 |
| FAM131C | 2.08440879 | 0.00656685 | family with sequence similarity 131 member C |
| TINAGL1 | 2.06627567 | 0.02635102 | tubulointerstitial nephritis antigen like 1 |
| TPSAB1 | 2.01435008 | 0.00052608 | tryptase alpha/beta 1 |
| CT69 | 1.99295841 | 0.01371807 | cancer/testis associated transcript 69 |
| APOBEC3A | 1.98386905 | 0.04644767 | apolipoprotein B mRNA editing enzyme catalytic subunit 3A |
| APOBEC3A_B | 1.98386905 | 0.04644767 | APOBEC3A and APOBEC3B deletion hybrid |
| THBS2 | 1.96562485 | 3.08E-05 | thrombospondin 2 |
| SLC15A1 | 1.95917817 | 3.73E-05 | solute carrier family 15 member 1 |
| CPA3 | 1.95722966 | 0.0275166 | carboxypeptidase A3 |
| DDIT4 | 1.95715628 | 1.22E-08 | DNA damage inducible transcript 4 |
| TNFSF9 | 1.92474579 | 0.0395841 | TNF superfamily member 9 |
| GFPT2 | 1.91463873 | 0.00728698 | glutamine-fructose-6-phosphate transaminase 2 |
| ZNF467 | 1.88594419 | 0.00490922 | zinc finger protein 467 |
| ICAM5 | 1.86868445 | 0.0006025 | intercellular adhesion molecule 5 |
| CFD | 1.85448073 | 0.02252689 | complement factor D |
| JCHAIN | 1.83913586 | 0.0354027 | joining chain of multimeric IgA and IgM |
| PRRX2 | 1.83646033 | 0.0005962 | paired related homeobox 2 |
| IL33 | 1.81467608 | 0.00549359 | interleukin 33 |
| CIB2 | 1.77749909 | 0.00375908 | calcium and integrin binding family member 2 |
| OASL | 1.73568984 | 0.00360969 | 2'-5'-oligoadenylate synthetase like |
| PNLIPRP3 | 1.7304124 | 0.04295777 | pancreatic lipase related protein 3 |
| KRT14 | 1.69933524 | 0.0183507 | keratin 14 |
| GCHFR | 1.69835866 | 0.0055048 | GTP cyclohydrolase I feedback regulator |
| EPOP | 1.68664846 | 1.13E-05 | elongin BC and polycomb repressive complex 2 associated protein |
| SORCS2 | 1.68175952 | 0.0001001 | sortilin related VPS10 domain containing receptor 2 |
| LIPK | 1.68164278 | 0.01197123 | lipase family member K |
| TNFRSF12A | 1.67821041 | 0.0004334 | TNF receptor superfamily member 12A |
| SHF | 1.6677323 | 9.40E-06 | Src homology 2 domain containing F |
| C4orf48 | 1.65597765 | 0.00039775 | chromosome 4 open reading frame 48 |
| CD22 | 1.64988913 | 0.04086023 | CD22 molecule |
| AGMAT | 1.64737904 | 0.00165364 | agmatinase |
| EEF1A2 | 1.63341587 | 0.03098446 | eukaryotic translation elongation factor 1 alpha 2 |
| FABP12 | 1.61234905 | 1.47E-05 | fatty acid binding protein 12 |
| MMP17 | 1.60282819 | 0.00388404 | matrix metallopeptidase 17 |
| TNFRSF18 | 1.60139592 | 0.00054056 | TNF receptor superfamily member 18 |
| EPHB2 | 1.57881403 | 0.0452255 | EPH receptor B2 |
| GALR2 | 1.57804361 | 0.03328204 | galanin receptor 2 |
| CDH16 | 1.57346401 | 0.00056832 | cadherin 16 |
| TFAP2E | 1.56372524 | 0.00539725 | transcription factor AP-2 epsilon |
| CAPN6 | 1.56090375 | 0.00476727 | calpain 6 |
| GGT1 | 1.56043591 | 0.0030344 | gamma-glutamyltransferase 1 |
| RRAD | 1.55091492 | 0.00346077 | RRAD, Ras related glycolysis inhibitor and calcium channel regulator |
| DZIP1 | 1.54702339 | 2.67E-10 | DAZ interacting zinc finger protein 1 |
| CDH17 | 1.53887368 | 0.00244279 | cadherin 17 |
| C1orf216 | 1.53689897 | 0.00021581 | chromosome 1 open reading frame 216 |
| NRARP | 1.5365936 | 4.44E-08 | NOTCH regulated ankyrin repeat protein |
| LORICRIN | 1.53431789 | 0.02301741 | loricrin cornified envelope precursor protein |
| CDKN1C | 1.51430104 | 0.00378792 | cyclin dependent kinase inhibitor 1C |
| SLC7A4 | 1.50904264 | 0.00967272 | solute carrier family 7 member 4 |
| ABCG1 | 1.48889145 | 0.03628997 | ATP binding cassette subfamily G member 1 |
| PHLDB2 | 1.4632024 | 2.34E-05 | pleckstrin homology like domain family B member 2 |
| RUFY4 | 1.45398534 | 0.01899852 | RUN and FYVE domain containing 4 |
| RARA-AS1 | 1.44205314 | 0.01802864 | RARA antisense RNA 1 |
| ACP7 | 1.42565806 | 0.0395841 | acid phosphatase 7, tartrate resistant (putative) |
| QPCT | 1.42113061 | 0.00539725 | glutaminyl-peptide cyclotransferase |
| LINC02762 | 1.42106814 | 0.00549359 | long intergenic non-protein coding RNA 2762 |
| CHRNA5 | 1.420674 | 0.00585687 | cholinergic receptor nicotinic alpha 5 subunit |
| FOSL1 | 1.41889951 | 0.04979434 | FOS like 1, AP-1 transcription factor subunit |
| HBEGF | 1.41015285 | 0.00031429 | heparin binding EGF like growth factor |
| TYMP | 1.40646112 | 4.19E-05 | thymidine phosphorylase |
| DLX5 | 1.39855884 | 0.0112712 | distal-less homeobox 5 |
| CHST6 | 1.38136693 | 0.01383149 | carbohydrate sulfotransferase 6 |
| CYP24A1 | 1.37513023 | 0.02301741 | cytochrome P450 family 24 subfamily A member 1 |
| RASL12 | 1.36862581 | 0.00966221 | RAS like family 12 |
| C1QTNF12 | 1.36151012 | 0.02523173 | C1q and TNF related 12 |
| RGS20 | 1.35672628 | 0.00290931 | regulator of G protein signaling 20 |
| KCTD17 | 1.35410405 | 0.04119652 | potassium channel tetramerization domain containing 17 |
| SEMA3B | 1.35053338 | 0.02347016 | semaphorin 3B |
| GLIS2 | 1.34587065 | 0.01418626 | GLIS family zinc finger 2 |
| ID1 | 1.34396609 | 0.0001782 | inhibitor of DNA binding 1, HLH protein |
| KLK1 | 1.34302411 | 0.01027621 | kallikrein 1 |
| IRX4 | 1.33479521 | 0.00010783 | iroquois homeobox 4 |
| EREG | 1.31730668 | 0.04941848 | epiregulin |
| MARCKSL1 | 1.30424438 | 0.00101719 | MARCKS like 1 |
| LTB | 1.30309847 | 0.0210383 | lymphotoxin beta |
| LINC01399 | 1.30196221 | 0.03608135 | long intergenic non-protein coding RNA 1399 |
| TPSB2 | 1.30068052 | 0.04279724 | tryptase beta 2 |
| VCAM1 | 1.29562374 | 0.04815002 | vascular cell adhesion molecule 1 |
| ADM2 | 1.29370337 | 0.00549359 | adrenomedullin 2 |
| ALDOA | 1.29268978 | 5.70E-08 | aldolase, fructose-bisphosphate A |
| PHLDA2 | 1.28888319 | 1.81E-05 | pleckstrin homology like domain family A member 2 |
| ABHD8 | 1.28290013 | 0.00515737 | abhydrolase domain containing 8 |
| GIPR | 1.26402392 | 0.01061369 | gastric inhibitory polypeptide receptor |
| DNAJB5 | 1.24943536 | 0.00850525 | DnaJ heat shock protein family (Hsp40) member B5 |
| CRLF1 | 1.23985263 | 0.01068851 | cytokine receptor like factor 1 |
| ARL2BP | 1.23409524 | 0.00101528 | ADP ribosylation factor like GTPase 2 binding protein |
| SUSD2 | 1.23236398 | 0.02150233 | sushi domain containing 2 |
| KRT16 | 1.23197219 | 0.01256912 | keratin 16 |
| THEMIS2 | 1.22527986 | 0.00747705 | thymocyte selection associated family member 2 |
| HAPLN3 | 1.2210555 | 0.00206534 | hyaluronan and proteoglycan link protein 3 |
| FER1L4 | 1.20304196 | 0.01858317 | fer-1 like family member 4 (pseudogene) |
| TMEM158 | 1.19949944 | 0.00694999 | transmembrane protein 158 |
| TMEM132A | 1.18930318 | 0.00214874 | transmembrane protein 132A |
| CSF2RB | 1.18031449 | 0.00244561 | colony stimulating factor 2 receptor subunit beta |
| SPACA6 | 1.179998 | 0.03599183 | sperm acrosome associated 6 |
| SEC14L2 | 1.16831977 | 0.04815002 | SEC14 like lipid binding 2 |
| SRXN1 | 1.16825501 | 0.00724165 | sulfiredoxin 1 |
| IQCA1 | 1.16813369 | 0.01907992 | IQ motif containing with AAA domain 1 |
| KIF3C | 1.15937144 | 0.02187455 | kinesin family member 3C |
| LARP6 | 1.15488818 | 0.03674623 | La ribonucleoprotein 6, translational regulator |
| NPAS1 | 1.14997679 | 0.01739764 | neuronal PAS domain protein 1 |
| HYAL1 | 1.14613742 | 0.00329347 | hyaluronidase 1 |
| PMEPA1 | 1.13724285 | 0.00065792 | prostate transmembrane protein, androgen induced 1 |
| AGTRAP | 1.13514796 | 0.00265457 | angiotensin II receptor associated protein |
| RPL39L | 1.13281319 | 0.00018021 | ribosomal protein L39 like |
| FAM229B | 1.12821273 | 0.00320268 | family with sequence similarity 229 member B |
| FSCN1 | 1.12757956 | 0.00359893 | fascin actin-bundling protein 1 |
| CTSC | 1.12601361 | 0.00048578 | cathepsin C |
| WNT10B | 1.11939496 | 0.00070803 | Wnt family member 10B |
| CEBPB | 1.11882013 | 1.68E-05 | CCAAT enhancer binding protein beta |
| SRRM3 | 1.1177525 | 0.0006491 | serine/arginine repetitive matrix 3 |
| EVA1B | 1.11557734 | 0.01750529 | eva-1 homolog B |
| IFITM1 | 1.11381365 | 0.00376206 | interferon induced transmembrane protein 1 |
| CLIC2 | 1.11181721 | 0.01131698 | chloride intracellular channel 2 |
| HOMER3 | 1.10937656 | 0.0002391 | homer scaffold protein 3 |
| SYNPO | 1.10472664 | 0.01442611 | synaptopodin |
| PDXP | 1.10095484 | 0.00832864 | pyridoxal phosphatase |
| TMEM9 | 1.09939293 | 0.00036047 | transmembrane protein 9 |
| CDH22 | 1.09504845 | 0.01504905 | cadherin 22 |
| OCEL1 | 1.08945256 | 0.00247363 | occludin/ELL domain containing 1 |
| MAPK8IP2 | 1.08663871 | 0.03454029 | mitogen-activated protein kinase 8 interacting protein 2 |
| SEMA6B | 1.08209094 | 0.03477532 | semaphorin 6B |
| ETV7 | 1.07713812 | 0.02635102 | ETS variant transcription factor 7 |
| CLCN4 | 1.07222614 | 0.04152653 | chloride voltage-gated channel 4 |
| GPR137B | 1.06695416 | 0.00539725 | G protein-coupled receptor 137B |
| KCNE3 | 1.06689276 | 0.00552807 | potassium voltage-gated channel subfamily E regulatory subunit 3 |
| ID3 | 1.06490465 | 1.61E-05 | inhibitor of DNA binding 3, HLH protein |
| ETV4 | 1.05787483 | 0.00189079 | ETS variant transcription factor 4 |
| CPNE7 | 1.05473966 | 0.0465199 | copine 7 |
| TTYH3 | 1.05180814 | 0.00290931 | tweety family member 3 |
| FGD2 | 1.0491278 | 0.02365396 | FYVE, RhoGEF and PH domain containing 2 |
| SERPINB12 | 1.04865896 | 0.04169663 | serpin family B member 12 |
| UBE2S | 1.04835082 | 0.0017271 | ubiquitin conjugating enzyme E2 S |
| SLC35E4 | 1.0482547 | 0.00314005 | solute carrier family 35 member E4 |
| IL1B | 1.04403551 | 0.01109131 | interleukin 1 beta |
| NXPH3 | 1.04400832 | 0.00593233 | neurexophilin 3 |
| KREMEN2 | 1.03488469 | 0.03852926 | kringle containing transmembrane protein 2 |
| TSPAN4 | 1.0320821 | 0.0055071 | tetraspanin 4 |
| MAPK12 | 1.02993876 | 0.02455753 | mitogen-activated protein kinase 12 |
| CBARP | 1.02920219 | 0.00754273 | CACN subunit beta associated regulatory protein |
| MSRB1 | 1.02747097 | 0.0001782 | methionine sulfoxide reductase B1 |
| FBXO2 | 1.01962728 | 6.80E-05 | F-box protein 2 |
| MFSD13A | 1.01883428 | 0.00629832 | major facilitator superfamily domain containing 13A |
| HES5 | 1.01850915 | 0.01946716 | hes family bHLH transcription factor 5 |
| CDR2L | 1.01677748 | 0.02779345 | cerebellar degeneration related protein 2 like |
| CEBPD | 1.01259969 | 2.34E-05 | CCAAT enhancer binding protein delta |
| EFHD2 | 1.01107644 | 0.01033807 | EF-hand domain family member D2 |
| FLNC | 1.0098768 | 0.02306964 | filamin C |
| PRR7 | 1.00320517 | 0.00967272 | proline rich 7, synaptic |
| LINC01671 | 0.99981879 | 0.04752155 | long intergenic non-protein coding RNA 1671 |
| HLA-B | 0.99600349 | 0.00546663 | major histocompatibility complex, class I, B |
| TRIB2 | 0.99253437 | 2.11E-06 | tribbles pseudokinase 2 |
| TMEM91 | 0.9914067 | 0.01752908 | transmembrane protein 91 |
| DDAH2 | 0.9843971 | 0.02232179 | dimethylarginine dimethylaminohydrolase 2 |
| IGF2BP3 | 0.97788441 | 0.0290521 | insulin like growth factor 2 mRNA binding protein 3 |
| STBD1 | 0.97608154 | 0.04636942 | starch binding domain 1 |
| ISYNA1 | 0.97607888 | 0.00080605 | inositol-3-phosphate synthase 1 |
| GPRIN1 | 0.97413382 | 0.04815002 | G protein regulated inducer of neurite outgrowth 1 |
| LMTK3 | 0.96962086 | 0.00972832 | lemur tyrosine kinase 3 |
| SLC15A3 | 0.96468569 | 0.04392042 | solute carrier family 15 member 3 |
| RPL23P8 | 0.95616158 | 0.01442963 | ribosomal protein L23 pseudogene 8 |
| ABTB1 | 0.94896866 | 0.02347097 | ankyrin repeat and BTB domain containing 1 |
| HMOX1 | 0.94830005 | 0.04603557 | heme oxygenase 1 |
| TGFA | 0.94821073 | 0.01865369 | transforming growth factor alpha |
| C1R | 0.94798989 | 0.00746042 | complement C1r |
| SRCIN1 | 0.94651931 | 0.04495098 | SRC kinase signaling inhibitor 1 |
| SIRPA | 0.94077899 | 1.10E-05 | signal regulatory protein alpha |
| FAM20C | 0.94065492 | 0.00549359 | FAM20C golgi associated secretory pathway kinase |
| SELENOM | 0.93986374 | 0.03385004 | selenoprotein M |
| FAM89A | 0.93943499 | 0.00476727 | family with sequence similarity 89 member A |
| DNASE1L3 | 0.93848336 | 0.01589939 | deoxyribonuclease 1 like 3 |
| SPINDOC | 0.93080181 | 0.03328204 | spindlin interactor and repressor of chromatin binding |
| TMEM238 | 0.92951968 | 1.50E-09 | transmembrane protein 238 |
| SLC4A3 | 0.92914555 | 0.03582297 | solute carrier family 4 member 3 |
| STING1 | 0.92856478 | 0.02253702 | stimulator of interferon response cGAMP interactor 1 |
| MTHFD1L | 0.92668632 | 0.00299019 | methylenetetrahydrofolate dehydrogenase (NADP+ dependent) 1 like |
| ARL4D | 0.92542662 | 0.00013282 | ADP ribosylation factor like GTPase 4D |
| LYPD5 | 0.92369481 | 0.0210383 | LY6/PLAUR domain containing 5 |
| PTPRZ1 | 0.92288814 | 0.00787734 | protein tyrosine phosphatase receptor type Z1 |
| ATP10A | 0.9201982 | 0.04815554 | ATPase phospholipid transporting 10A (putative) |
| CDH26 | 0.90361875 | 0.04795364 | cadherin 26 |
| IER5L | 0.90288667 | 0.0055048 | immediate early response 5 like |
| C1S | 0.90154398 | 0.02446858 | complement C1s |
| OSBP2 | -0.9074916 | 0.00966221 | oxysterol binding protein 2 |
| KIF21A | -0.9083152 | 0.02218322 | kinesin family member 21A |
| LRRK2 | -0.9186395 | 0.00832823 | leucine rich repeat kinase 2 |
| FCGR2B | -0.9369545 | 0.01535053 | Fc fragment of IgG receptor IIb |
| DTX4 | -0.9370962 | 0.0039768 | deltex E3 ubiquitin ligase 4 |
| GOLGA8B | -0.9445191 | 0.00381781 | golgin A8 family member B |
| DEPTOR | -0.9547372 | 0.0055048 | DEP domain containing MTOR interacting protein |
| SPON2 | -0.9586442 | 0.01436262 | spondin 2 |
| C9orf152 | -0.9658117 | 0.00333339 | chromosome 9 open reading frame 152 |
| CPED1 | -0.9680865 | 0.02392406 | cadherin like and PC-esterase domain containing 1 |
| LINC00649 | -0.9706543 | 0.04800289 | long intergenic non-protein coding RNA 649 |
| SPTLC3 | -0.9713119 | 0.00299123 | serine palmitoyltransferase long chain base subunit 3 |
| CMYA5 | -0.9753426 | 0.01027621 | cardiomyopathy associated 5 |
| HBB | -0.9778722 | 0.02124055 | hemoglobin subunit beta |
| MBNL1-AS1 | -0.980036 | 0.01293497 | MBNL1 antisense RNA 1 |
| KEL | -0.9878714 | 0.002004 | Kell metallo-endopeptidase (Kell blood group) |
| COL21A1 | -0.9962913 | 0.03166537 | collagen type XXI alpha 1 chain |
| FAM171A1 | -1.0009214 | 6.20E-07 | family with sequence similarity 171 member A1 |
| B3GAT2 | -1.0039528 | 0.04825404 | beta-1,3-glucuronyltransferase 2 |
| C2orf92 | -1.0171409 | 0.02145963 | chromosome 2 open reading frame 92 |
| GPT | -1.0183373 | 0.00412079 | glutamic--pyruvic transaminase |
| TMEM47 | -1.0218368 | 0.01433591 | transmembrane protein 47 |
| ZNF555 | -1.0233633 | 0.01574484 | zinc finger protein 555 |
| ZNF701 | -1.0246168 | 0.00047222 | zinc finger protein 701 |
| P2RX7 | -1.0280269 | 0.00498644 | purinergic receptor P2X 7 |
| GUCY1A2 | -1.0338716 | 0.02387682 | guanylate cyclase 1 soluble subunit alpha 2 |
| BFSP1 | -1.0492278 | 0.00123989 | beaded filament structural protein 1 |
| RNF152 | -1.0504544 | 0.00403488 | ring finger protein 152 |
| STRADB | -1.0544532 | 0.01622059 | STE20 related adaptor beta |
| CD1C | -1.0698896 | 0.00039775 | CD1c molecule |
| KLB | -1.0700613 | 0.00069546 | klotho beta |
| MASP2 | -1.0769 | 0.00135491 | MBL associated serine protease 2 |
| SLC15A2 | -1.0842825 | 0.00488451 | solute carrier family 15 member 2 |
| ZNF337-AS1 | -1.0848239 | 0.00080605 | ZNF337 antisense RNA 1 |
| RTN1 | -1.0891512 | 0.0183507 | reticulon 1 |
| SLC16A2 | -1.1018257 | 0.00168518 | solute carrier family 16 member 2 |
| MROH8 | -1.1078694 | 0.01750529 | maestro heat like repeat family member 8 |
| CCR2 | -1.1136211 | 0.00557417 | C-C motif chemokine receptor 2 |
| TSPAN12 | -1.1257164 | 0.00172303 | tetraspanin 12 |
| FOSL2-AS1 | -1.1414974 | 0.03557556 | FOSL2 antisense RNA 1 |
| LMCD1 | -1.1450022 | 2.21E-06 | LIM and cysteine rich domains 1 |
| RDH5 | -1.1602831 | 0.01941836 | retinol dehydrogenase 5 |
| IGSF11 | -1.1614502 | 0.01578939 | immunoglobulin superfamily member 11 |
| CYP2F1 | -1.1743337 | 0.00299019 | cytochrome P450 family 2 subfamily F member 1 |
| XCR1 | -1.1926555 | 0.04534275 | X-C motif chemokine receptor 1 |
| NAALADL2 | -1.1967437 | 0.00867276 | N-acetylated alpha-linked acidic dipeptidase like 2 |
| FAM189A2 | -1.2022772 | 2.21E-06 | family with sequence similarity 189 member A2 |
| NELL2 | -1.2050626 | 0.02326957 | neural EGFL like 2 |
| ADCY5 | -1.2107697 | 0.04677732 | adenylate cyclase 5 |
| AMOT | -1.2255452 | 6.11E-05 | angiomotin |
| ALDH1A1 | -1.2263908 | 0.03533169 | aldehyde dehydrogenase 1 family member A1 |
| CFTR | -1.2346198 | 0.00556754 | CF transmembrane conductance regulator |
| RND3 | -1.2430825 | 0.03533169 | Rho family GTPase 3 |
| SLC2A12 | -1.2512746 | 0.0001001 | solute carrier family 2 member 12 |
| PDGFD | -1.2557241 | 0.00553874 | platelet derived growth factor D |
| ABI3BP | -1.2587555 | 0.01941836 | ABI family member 3 binding protein |
| CLEC5A | -1.2693106 | 0.00360348 | C-type lectin domain containing 5A |
| C8orf82 | -1.305477 | 6.46E-05 | chromosome 8 open reading frame 82 |
| SLC26A2 | -1.3483835 | 3.90E-06 | solute carrier family 26 member 2 |
| MIR100HG | -1.3695583 | 0.01750529 | mir-100-let-7a-2-mir-125b-1 cluster host gene |
| FSTL4 | -1.3779279 | 0.01452627 | follistatin like 4 |
| KCNK13 | -1.3870182 | 0.04119652 | potassium two pore domain channel subfamily K member 13 |
| DUOX2 | -1.3955855 | 0.00101377 | dual oxidase 2 |
| CX3CR1 | -1.4142718 | 0.0028082 | C-X3-C motif chemokine receptor 1 |
| CLEC4F | -1.4164544 | 0.00425833 | C-type lectin domain family 4 member F |
| STXBP6 | -1.4276398 | 0.00237008 | syntaxin binding protein 6 |
| SLC10A5 | -1.4424188 | 0.03880825 | solute carrier family 10 member 5 |
| KCNMA1 | -1.4709906 | 0.00549359 | potassium calcium-activated channel subfamily M alpha 1 |
| FRAS1 | -1.4981776 | 1.13E-05 | Fraser extracellular matrix complex subunit 1 |
| NEXMIF | -1.5080529 | 0.04253672 | neurite extension and migration factor |
| CRISP3 | -1.5223288 | 0.01145014 | cysteine rich secretory protein 3 |
| PEG3 | -1.5434233 | 0.02580608 | paternally expressed 3 |
| NRG4 | -1.5711698 | 0.00797936 | neuregulin 4 |
| FETUB | -1.5731328 | 0.02232179 | fetuin B |
| SLCO2B1 | -1.5745591 | 0.00489458 | solute carrier organic anion transporter family member 2B1 |
| CTNNAL1 | -1.5875462 | 1.93E-05 | catenin alpha like 1 |
| LRRC7 | -1.6426793 | 0.01075741 | leucine rich repeat containing 7 |
| IGFBP3 | -1.6738753 | 0.02615222 | insulin like growth factor binding protein 3 |
| MTUS2 | -1.7363863 | 0.00244561 | microtubule associated scaffold protein 2 |
| GNA14 | -1.737563 | 0.00141603 | G protein subunit alpha 14 |
| FLG2 | -1.7578069 | 0.04979434 | filaggrin family member 2 |
| PDE1A | -1.7682378 | 0.00101528 | phosphodiesterase 1A |
| TMEM63C | -1.7715649 | 7.18E-10 | transmembrane protein 63C |
| PDE3A | -1.7798467 | 0.0043772 | phosphodiesterase 3A |
| WFDC21P | -1.7802765 | 0.00088762 | WAP four-disulfide core domain 21, pseudogene |
| CYP4F35P | -1.7915921 | 2.34E-05 | cytochrome P450 family 4 subfamily F member 35, pseudogene |
| LRRC14 | -1.8097653 | 0.0006025 | leucine rich repeat containing 14 |
| PDE6A | -1.8245613 | 5.38E-06 | phosphodiesterase 6A |
| OXGR1 | -1.8250759 | 0.00250306 | oxoglutarate receptor 1 |
| ALCAM | -1.8266032 | 0.00302014 | activated leukocyte cell adhesion molecule |
| SBSPON | -1.9433824 | 0.00130479 | somatomedin B and thrombospondin type 1 domain containing |
| ETNK2 | -1.9605602 | 1.72E-08 | ethanolamine kinase 2 |
| FMO9P | -1.9687626 | 0.0102173 | flavin containing dimethylaniline monoxygenase 9, pseudogene |
| NOG | -1.9968863 | 1.22E-06 | noggin |
| COLEC12 | -2.0030739 | 0.00116886 | collectin subfamily member 12 |
| IRAG2 | -2.0246095 | 0.0107759 | inositol 1,4,5-triphosphate receptor associated 2 |
| EDN3 | -2.0517851 | 0.04695704 | endothelin 3 |
| LINC01820 | -2.0522371 | 0.01199496 | long intergenic non-protein coding RNA 1820 |
| LINC01587 | -2.0782311 | 1.61E-05 | long intergenic non-protein coding RNA 1587 |
| DUOXA2 | -2.11366 | 0.0112712 | dual oxidase maturation factor 2 |
| ISM1 | -2.1715049 | 0.00069546 | isthmin 1 |
| FOSB | -2.1967611 | 0.00792424 | FosB proto-oncogene, AP-1 transcription factor subunit |
| RECQL4 | -2.228269 | 0.00030557 | RecQ like helicase 4 |
| REN | -2.2490066 | 0.00054056 | renin |
| LOC101929106 | -2.3287449 | 0.00042668 | uncharacterized LOC101929106 |
| RNASE7 | -2.339611 | 0.00369856 | ribonuclease A family member 7 |
| CRYM | -2.3700753 | 0.00206534 | crystallin mu |
| CRISP2 | -2.3806269 | 0.03310329 | cysteine rich secretory protein 2 |
| HERC2P3 | -2.4679538 | 0.00165583 | HERC2 pseudogene 3 |
| MAMDC2 | -2.4801794 | 3.36E-09 | MAM domain containing 2 |
| FGF14 | -2.6329279 | 1.13E-05 | fibroblast growth factor 14 |
| MUC22 | -2.7136453 | 0.02218322 | mucin 22 |
| DPYSL3 | -2.7777521 | 1.66E-11 | dihydropyrimidinase like 3 |
| APLN | -2.9999466 | 0.03328204 | apelin |
| KRTAP3-2 | -3.0786482 | 0.00024548 | keratin associated protein 3-2 |
| SLITRK5 | -3.4750229 | 6.20E-07 | SLIT and NTRK like family member 5 |
| LINC02099 | -3.5228299 | 0.03037824 | long intergenic non-protein coding RNA 2099 |
| DMRT3 | -3.6539769 | 0.01701611 | doublesex and mab-3 related transcription factor 3 |
| DKK2 | -4.1176534 | 0.00580298 | dickkopf WNT signaling pathway inhibitor 2 |
| MTRNR2L1 | -4.8855059 | 0.00290931 | MT-RNR2 like 1 |

**Supplementary Table 6. Genes differentially expressed in proximal esophagus of type 2 achalasia vs. healthy control**

| **Gene Symbol** | **Log2 Fold Change** | **FDR Adj p Value** | **Description** |
| --- | --- | --- | --- |
| NEFM | 4.37502449 | 0.00026489 | neurofilament medium chain |
| LCE3A | 3.44773146 | 0.00305979 | late cornified envelope 3A |
| NEFL | 3.44357821 | 0.00144588 | neurofilament light chain |
| GLDC | 2.57695582 | 0.00021941 | glycine decarboxylase |
| CCER2 | 2.53963628 | 0.02582585 | coiled-coil glutamate rich protein 2 |
| LORICRIN | 2.51111107 | 0.0146686 | loricrin cornified envelope precursor protein |
| TPSD1 | 2.45676331 | 0.00787995 | tryptase delta 1 |
| ANK1 | 2.39756888 | 0.00073109 | ankyrin 1 |
| RN7SL2 | 2.39548848 | 1.39E-06 | RNA component of signal recognition particle 7SL2 |
| GFPT2 | 2.19442751 | 0.0007196 | glutamine-fructose-6-phosphate transaminase 2 |
| RRAD | 2.13198531 | 1.05E-05 | RRAD, Ras related glycolysis inhibitor and calcium channel regulator |
| MARCO | 2.11601009 | 5.83E-08 | macrophage receptor with collagenous structure |
| APOE | 2.06144812 | 0.01869954 | apolipoprotein E |
| KRT1 | 2.01246349 | 0.04720479 | keratin 1 |
| KRTDAP | 2.00539877 | 0.03522077 | keratinocyte differentiation associated protein |
| TPSAB1 | 1.91839794 | 6.51E-09 | tryptase alpha/beta 1 |
| C4orf48 | 1.89979026 | 8.75E-06 | chromosome 4 open reading frame 48 |
| PNLIPRP3 | 1.87153992 | 0.00520689 | pancreatic lipase related protein 3 |
| CDH16 | 1.85096186 | 0.00045659 | cadherin 16 |
| PALM | 1.8363705 | 0.01304322 | paralemmin |
| EPOP | 1.79524022 | 1.07E-07 | elongin BC and polycomb repressive complex 2 associated protein |
| ARC | 1.75522378 | 0.02959912 | activity regulated cytoskeleton associated protein |
| HES5 | 1.73783065 | 1.07E-07 | hes family bHLH transcription factor 5 |
| TSPAN10 | 1.71692775 | 0.01867961 | tetraspanin 10 |
| PI3 | 1.71188485 | 3.39E-05 | peptidase inhibitor 3 |
| CIDEA | 1.68912962 | 0.03965392 | cell death inducing DFFA like effector a |
| DUSP2 | 1.6818611 | 0.02165092 | dual specificity phosphatase 2 |
| TMEM45A | 1.64750274 | 3.50E-05 | transmembrane protein 45A |
| DDIT4 | 1.64713205 | 4.88E-07 | DNA damage inducible transcript 4 |
| SLC29A4 | 1.6359115 | 0.00197762 | solute carrier family 29 member 4 |
| MYOZ1 | 1.61829103 | 0.03715349 | myozenin 1 |
| FAM131C | 1.59809515 | 0.0013615 | family with sequence similarity 131 member C |
| ICAM5 | 1.58781756 | 0.00309661 | intercellular adhesion molecule 5 |
| TINAGL1 | 1.58748619 | 0.00199018 | tubulointerstitial nephritis antigen like 1 |
| MMP9 | 1.581105 | 0.00610349 | matrix metallopeptidase 9 |
| RASL12 | 1.56312483 | 0.00026189 | RAS like family 12 |
| KLHDC7B | 1.53596343 | 0.02274762 | kelch domain containing 7B |
| STRA6 | 1.52726599 | 0.03800193 | signaling receptor and transporter of retinol STRA6 |
| PRRX2 | 1.52704576 | 0.0005153 | paired related homeobox 2 |
| CPA3 | 1.52318784 | 0.00093813 | carboxypeptidase A3 |
| PGBD5 | 1.52258262 | 0.00057985 | piggyBac transposable element derived 5 |
| FAM25A | 1.51073134 | 0.02909339 | family with sequence similarity 25 member A |
| TFAP2E | 1.48255195 | 0.01564423 | transcription factor AP-2 epsilon |
| VSIG8 | 1.48099775 | 0.01991455 | V-set and immunoglobulin domain containing 8 |
| CARD17 | 1.47074971 | 0.00561434 | caspase recruitment domain family member 17 |
| KREMEN2 | 1.46496735 | 0.00085552 | kringle containing transmembrane protein 2 |
| DZIP1 | 1.44660059 | 4.47E-09 | DAZ interacting zinc finger protein 1 |
| RDH16 | 1.43019783 | 0.02206912 | retinol dehydrogenase 16 |
| TNFRSF18 | 1.42903284 | 0.00070823 | TNF receptor superfamily member 18 |
| PTGER1 | 1.4139328 | 0.00024864 | prostaglandin E receptor 1 |
| ANGPTL4 | 1.40661954 | 0.00169813 | angiopoietin like 4 |
| CAPN6 | 1.38209719 | 0.00100527 | calpain 6 |
| TNFRSF12A | 1.37915622 | 4.46E-06 | TNF receptor superfamily member 12A |
| ALDOA | 1.36603945 | 1.76E-06 | aldolase, fructose-bisphosphate A |
| C1orf216 | 1.36303424 | 0.00086948 | chromosome 1 open reading frame 216 |
| OASL | 1.35978123 | 0.00144838 | 2'-5'-oligoadenylate synthetase like |
| IER5L | 1.33757819 | 2.48E-05 | immediate early response 5 like |
| SDSL | 1.33485373 | 0.00216881 | serine dehydratase like |
| SUSD2 | 1.328099 | 8.33E-05 | sushi domain containing 2 |
| MMP17 | 1.32590178 | 0.00218741 | matrix metallopeptidase 17 |
| NRARP | 1.32304048 | 0.00309212 | NOTCH regulated ankyrin repeat protein |
| SHF | 1.31512608 | 0.00112366 | Src homology 2 domain containing F |
| LOC100133091 | 1.31081199 | 0.03200413 | uncharacterized LOC100133091 |
| PANX2 | 1.30763838 | 0.01302468 | pannexin 2 |
| FABP12 | 1.29716734 | 0.00026189 | fatty acid binding protein 12 |
| GGT1 | 1.29513476 | 0.00186185 | gamma-glutamyltransferase 1 |
| LINC01497 | 1.29199909 | 0.0246222 | long intergenic non-protein coding RNA 1497 |
| MFSD6L | 1.26295713 | 0.00386645 | major facilitator superfamily domain containing 6 like |
| TPSB2 | 1.26079327 | 0.0134424 | tryptase beta 2 |
| SEMA3B | 1.25820589 | 6.21E-07 | semaphorin 3B |
| CDKN1C | 1.257576 | 0.00011151 | cyclin dependent kinase inhibitor 1C |
| CARD18 | 1.25208941 | 0.00940045 | caspase recruitment domain family member 18 |
| SYT12 | 1.24619958 | 0.02950114 | synaptotagmin 12 |
| LINC01671 | 1.24203638 | 0.00602485 | long intergenic non-protein coding RNA 1671 |
| TMEM158 | 1.23732055 | 0.00228855 | transmembrane protein 158 |
| TEPP | 1.23481603 | 0.03649384 | testis, prostate and placenta expressed |
| CIB2 | 1.23377765 | 0.00510289 | calcium and integrin binding family member 2 |
| SNORD104 | 1.22928574 | 0.0005322 | small nucleolar RNA, C/D box 104 |
| GCHFR | 1.2233468 | 0.00091054 | GTP cyclohydrolase I feedback regulator |
| C20orf204 | 1.22302002 | 0.00101497 | chromosome 20 open reading frame 204 |
| ARL2BP | 1.2165668 | 0.00019156 | ADP ribosylation factor like GTPase 2 binding protein |
| PLA2G4D | 1.21064525 | 0.02646973 | phospholipase A2 group IVD |
| SMTNL2 | 1.20924425 | 0.01091664 | smoothelin like 2 |
| CFD | 1.2051278 | 0.01978039 | complement factor D |
| RARRES2 | 1.20207773 | 0.01184001 | retinoic acid receptor responder 2 |
| ABHD8 | 1.19291462 | 4.90E-07 | abhydrolase domain containing 8 |
| SORCS2 | 1.19189098 | 0.00107702 | sortilin related VPS10 domain containing receptor 2 |
| ATP13A5 | 1.18905136 | 0.01770494 | ATPase 13A5 |
| MYH13 | 1.18772173 | 0.029793 | myosin heavy chain 13 |
| HEPH | 1.18499014 | 0.03200413 | hephaestin |
| ALOX15 | 1.18456081 | 0.00741288 | arachidonate 15-lipoxygenase |
| NXPH4 | 1.18363781 | 0.00124535 | neurexophilin 4 |
| MAPK8IP2 | 1.18319791 | 0.01235906 | mitogen-activated protein kinase 8 interacting protein 2 |
| NPW | 1.17991572 | 0.01055776 | neuropeptide W |
| ID1 | 1.17763341 | 0.00011125 | inhibitor of DNA binding 1, HLH protein |
| EVA1B | 1.17645187 | 2.72E-09 | eva-1 homolog B |
| AGMAT | 1.17565229 | 0.04407159 | agmatinase |
| ZNF467 | 1.17140163 | 0.00132142 | zinc finger protein 467 |
| IRX4 | 1.16285926 | 0.00081083 | iroquois homeobox 4 |
| HOMER3 | 1.16261399 | 1.91E-06 | homer scaffold protein 3 |
| LINC02762 | 1.15918883 | 0.02005887 | long intergenic non-protein coding RNA 2762 |
| EEF1A2 | 1.14882721 | 0.01495068 | eukaryotic translation elongation factor 1 alpha 2 |
| RARA-AS1 | 1.14621566 | 0.01061068 | RARA antisense RNA 1 |
| PRR7 | 1.14496014 | 2.97E-05 | proline rich 7, synaptic |
| THBS2 | 1.14372649 | 0.00550285 | thrombospondin 2 |
| WNT10B | 1.14004967 | 0.0371794 | Wnt family member 10B |
| POU3F1 | 1.13051767 | 0.00016281 | POU class 3 homeobox 1 |
| CHST6 | 1.1293422 | 0.0181845 | carbohydrate sulfotransferase 6 |
| H1-2 | 1.12428722 | 0.0146686 | H1.2 linker histone, cluster member |
| SEZ6L2 | 1.12299064 | 0.04373238 | seizure related 6 homolog like 2 |
| HSH2D | 1.11946233 | 0.01495068 | hematopoietic SH2 domain containing |
| PDXP | 1.11911189 | 0.00070823 | pyridoxal phosphatase |
| PROB1 | 1.11359153 | 9.80E-05 | proline rich basic protein 1 |
| NYAP1 | 1.11132289 | 0.01021532 | neuronal tyrosine phosphorylated phosphoinositide-3-kinase adaptor 1 |
| RPL23P8 | 1.11024847 | 0.00121301 | ribosomal protein L23 pseudogene 8 |
| DLX3 | 1.09537708 | 0.00206262 | distal-less homeobox 3 |
| COL16A1 | 1.09043711 | 1.00E-06 | collagen type XVI alpha 1 chain |
| CRLF1 | 1.08711955 | 0.04135706 | cytokine receptor like factor 1 |
| COL13A1 | 1.085765 | 0.02162708 | collagen type XIII alpha 1 chain |
| ISYNA1 | 1.08340874 | 0.00022024 | inositol-3-phosphate synthase 1 |
| RUFY4 | 1.07805579 | 0.02240218 | RUN and FYVE domain containing 4 |
| C1QTNF12 | 1.07701501 | 0.033858 | C1q and TNF related 12 |
| TYMP | 1.06973337 | 5.34E-05 | thymidine phosphorylase |
| SLC7A4 | 1.06601221 | 0.00508605 | solute carrier family 7 member 4 |
| ID3 | 1.06576363 | 3.27E-07 | inhibitor of DNA binding 3, HLH protein |
| DRAP1 | 1.06550868 | 0.00052787 | DR1 associated protein 1 |
| PGF | 1.06340453 | 0.00107702 | placental growth factor |
| SLC45A4 | 1.06184944 | 2.97E-05 | solute carrier family 45 member 4 |
| DNAJB5 | 1.05984428 | 0.00086461 | DnaJ heat shock protein family (Hsp40) member B5 |
| KRT16 | 1.05902655 | 0.0016157 | keratin 16 |
| PTPRU | 1.05806045 | 0.00036438 | protein tyrosine phosphatase receptor type U |
| TMEM176A | 1.05407257 | 0.04711845 | transmembrane protein 176A |
| GLIS2 | 1.05396838 | 0.00182139 | GLIS family zinc finger 2 |
| FSCN1 | 1.05330623 | 0.0002492 | fascin actin-bundling protein 1 |
| ABCG1 | 1.0530473 | 0.02181257 | ATP binding cassette subfamily G member 1 |
| TPM2 | 1.05176293 | 0.00266813 | tropomyosin 2 |
| TMEM238 | 1.04870154 | 1.66E-14 | transmembrane protein 238 |
| RAB17 | 1.04467207 | 0.00520689 | RAB17, member RAS oncogene family |
| RBP1 | 1.03945083 | 0.04186156 | retinol binding protein 1 |
| PLTP | 1.0324959 | 0.04015703 | phospholipid transfer protein |
| MLF1 | 1.02893419 | 0.01636535 | myeloid leukemia factor 1 |
| SLC2A6 | 1.02393196 | 0.0372341 | solute carrier family 2 member 6 |
| SPAG4 | 1.01997386 | 0.00280973 | sperm associated antigen 4 |
| SLC12A8 | 1.01572209 | 0.00767024 | solute carrier family 12 member 8 |
| WTIP | 1.0147938 | 2.12E-06 | WT1 interacting protein |
| AKR1B10 | 1.01326099 | 0.00012102 | aldo-keto reductase family 1 member B10 |
| SFXN5 | 1.01124671 | 8.60E-05 | sideroflexin 5 |
| SYT7 | 1.00989921 | 0.00073792 | synaptotagmin 7 |
| RPL39L | 1.0098451 | 0.00034673 | ribosomal protein L39 like |
| SLC16A3 | 1.00694456 | 0.0029781 | solute carrier family 16 member 3 |
| ABCA7 | 1.00394437 | 2.97E-06 | ATP binding cassette subfamily A member 7 |
| DNASE1L2 | 1.00230102 | 0.02467879 | deoxyribonuclease 1 like 2 |
| SRXN1 | 1.00201675 | 0.00776822 | sulfiredoxin 1 |
| THEMIS2 | 1.00143161 | 0.00218046 | thymocyte selection associated family member 2 |
| AK1 | 1.00097809 | 0.00013188 | adenylate kinase 1 |
| PTAFR | 0.99752434 | 0.00436922 | platelet activating factor receptor |
| SLC15A1 | 0.99206923 | 0.00419078 | solute carrier family 15 member 1 |
| SLC5A10 | 0.99135804 | 0.02287327 | solute carrier family 5 member 10 |
| FBXO2 | 0.99065731 | 4.03E-06 | F-box protein 2 |
| NGFR | 0.98633576 | 0.00418488 | nerve growth factor receptor |
| GPR160 | 0.98313615 | 0.03344982 | G protein-coupled receptor 160 |
| CEBPB | 0.97869837 | 0.00045659 | CCAAT enhancer binding protein beta |
| CRABP2 | 0.97442444 | 2.74E-06 | cellular retinoic acid binding protein 2 |
| CPNE7 | 0.97168444 | 0.02901231 | copine 7 |
| ARL4D | 0.97147424 | 0.00026971 | ADP ribosylation factor like GTPase 4D |
| LARP6 | 0.96940625 | 0.02146972 | La ribonucleoprotein 6, translational regulator |
| QPCT | 0.96147046 | 0.04374899 | glutaminyl-peptide cyclotransferase |
| SLC22A18AS | 0.96024878 | 0.03419448 | solute carrier family 22 member 18 antisense |
| TGFB1 | 0.95908483 | 0.04876369 | transforming growth factor beta 1 |
| DDAH2 | 0.95581116 | 0.00309212 | dimethylarginine dimethylaminohydrolase 2 |
| PHLDB2 | 0.95232356 | 0.02370459 | pleckstrin homology like domain family B member 2 |
| SIRPA | 0.95044861 | 1.24E-07 | signal regulatory protein alpha |
| LTB | 0.9469063 | 0.04336818 | lymphotoxin beta |
| TSPAN4 | 0.94531038 | 8.21E-07 | tetraspanin 4 |
| ASGR1 | 0.94360349 | 0.04154631 | asialoglycoprotein receptor 1 |
| CHI3L1 | 0.94337993 | 0.00201354 | chitinase 3 like 1 |
| CEBPD | 0.94335922 | 0.00018019 | CCAAT enhancer binding protein delta |
| LDHD | 0.94314065 | 2.43E-06 | lactate dehydrogenase D |
| SH2B2 | 0.9410128 | 0.0017868 | SH2B adaptor protein 2 |
| STBD1 | 0.93996382 | 0.02357578 | starch binding domain 1 |
| PDF | 0.93401735 | 0.0181845 | peptide deformylase, mitochondrial |
| RGS20 | 0.92759365 | 0.00866053 | regulator of G protein signaling 20 |
| SLC35E4 | 0.92510843 | 0.00246871 | solute carrier family 35 member E4 |
| HAPLN3 | 0.91982159 | 1.68E-05 | hyaluronan and proteoglycan link protein 3 |
| CBARP | 0.91496132 | 0.00018884 | CACN subunit beta associated regulatory protein |
| LRFN4 | 0.91339239 | 0.00190388 | leucine rich repeat and fibronectin type III domain containing 4 |
| NINJ1 | 0.91047246 | 0.00012502 | ninjurin 1 |
| TMEM38A | 0.9051728 | 0.00187182 | transmembrane protein 38A |
| GPR153 | 0.90150102 | 0.00175344 | G protein-coupled receptor 153 |
| IQCA1 | 0.90107591 | 0.0384132 | IQ motif containing with AAA domain 1 |
| CTU1 | 0.90047172 | 2.29E-07 | cytosolic thiouridylase subunit 1 |
| ZNF528-AS1 | -0.9021901 | 0.00458118 | ZNF528 antisense RNA 1 |
| FCGR2B | -0.9043849 | 0.00093813 | Fc fragment of IgG receptor IIb |
| RSKR | -0.9052298 | 0.00108782 | ribosomal protein S6 kinase related |
| KCNMA1 | -0.9123126 | 0.03929435 | potassium calcium-activated channel subfamily M alpha 1 |
| PLCB1 | -0.9139739 | 0.01875677 | phospholipase C beta 1 |
| LOC100128494 | -0.9203135 | 0.00600773 | uncharacterized LOC100128494 |
| EPHA1-AS1 | -0.9255407 | 0.02038759 | EPHA1 antisense RNA 1 |
| MFSD4A | -0.9320923 | 0.02966243 | major facilitator superfamily domain containing 4A |
| ARNTL | -0.9399972 | 0.01077706 | aryl hydrocarbon receptor nuclear translocator like |
| GVINP1 | -0.9403691 | 0.00326601 | GTPase, very large interferon inducible pseudogene 1 |
| SAMD9 | -0.9504821 | 0.01790371 | sterile alpha motif domain containing 9 |
| SNX31 | -0.9562571 | 0.01214183 | sorting nexin 31 |
| TTN | -0.9599284 | 0.00520595 | titin |
| CRISP3 | -0.9675492 | 0.03778014 | cysteine rich secretory protein 3 |
| TCN1 | -0.9685187 | 0.02900325 | transcobalamin 1 |
| CX3CR1 | -0.9703621 | 0.01508779 | C-X3-C motif chemokine receptor 1 |
| ACSM3 | -0.9744605 | 0.0022876 | acyl-CoA synthetase medium chain family member 3 |
| FRAS1 | -0.9745921 | 0.00021941 | Fraser extracellular matrix complex subunit 1 |
| PIK3CG | -0.9804073 | 0.00105559 | phosphatidylinositol-4,5-bisphosphate 3-kinase catalytic subunit gamma |
| TSPAN12 | -0.9815259 | 1.83E-05 | tetraspanin 12 |
| C8orf82 | -0.982025 | 0.00023173 | chromosome 8 open reading frame 82 |
| C2orf92 | -0.9825963 | 0.00225349 | chromosome 2 open reading frame 92 |
| SFRP1 | -0.9856678 | 0.00850771 | secreted frizzled related protein 1 |
| NCALD | -0.9942089 | 0.03726823 | neurocalcin delta |
| GCNT1 | -0.9975587 | 0.00724006 | glucosaminyl (N-acetyl) transferase 1 |
| NELL2 | -1.0002515 | 0.02382344 | neural EGFL like 2 |
| C12orf56 | -1.0029465 | 0.01071465 | chromosome 12 open reading frame 56 |
| JMJD7 | -1.0093894 | 0.0426593 | jumonji domain containing 7 |
| ANKRD36 | -1.0121423 | 0.01628554 | ankyrin repeat domain 36 |
| CCR2 | -1.0208492 | 0.00682993 | C-C motif chemokine receptor 2 |
| LOC100128398 | -1.0368363 | 0.00053149 | uncharacterized LOC100128398 |
| COL21A1 | -1.0372228 | 0.00442463 | collagen type XXI alpha 1 chain |
| RNF152 | -1.0422865 | 0.00026636 | ring finger protein 152 |
| TLR6 | -1.0525962 | 0.00218741 | toll like receptor 6 |
| HNMT | -1.0548395 | 0.00030718 | histamine N-methyltransferase |
| LAMA2 | -1.0562835 | 0.00407727 | laminin subunit alpha 2 |
| SLAMF6 | -1.057869 | 0.01081821 | SLAM family member 6 |
| YOD1 | -1.0601597 | 0.00267097 | YOD1 deubiquitinase |
| SLC8A1 | -1.0627704 | 0.00114056 | solute carrier family 8 member A1 |
| AMOT | -1.0635459 | 3.50E-05 | angiomotin |
| CPNE4 | -1.0682519 | 0.01158933 | copine 4 |
| CCDC127 | -1.0757943 | 0.001306 | coiled-coil domain containing 127 |
| CFAP91 | -1.0772919 | 0.01650948 | cilia and flagella associated protein 91 |
| MTUS2 | -1.0893441 | 0.030557 | microtubule associated scaffold protein 2 |
| BMS1P1 | -1.0903419 | 0.00035223 | BMS1 pseudogene 1 |
| PTGER3 | -1.0905481 | 0.03991315 | prostaglandin E receptor 3 |
| TRMT9B | -1.0959246 | 0.01067937 | tRNA methyltransferase 9B (putative) |
| CFTR | -1.1035966 | 0.00224934 | CF transmembrane conductance regulator |
| RHOBTB3 | -1.1052053 | 0.01960085 | Rho related BTB domain containing 3 |
| CLEC4F | -1.1154746 | 0.01900745 | C-type lectin domain family 4 member F |
| XCR1 | -1.1211451 | 0.01033225 | X-C motif chemokine receptor 1 |
| GPRIN3 | -1.1343111 | 5.70E-06 | GPRIN family member 3 |
| STXBP6 | -1.1393333 | 0.00358619 | syntaxin binding protein 6 |
| CDK14 | -1.1524544 | 0.01169995 | cyclin dependent kinase 14 |
| ANKS1B | -1.153035 | 0.0104908 | ankyrin repeat and sterile alpha motif domain containing 1B |
| IGFBP3 | -1.15833 | 0.04676193 | insulin like growth factor binding protein 3 |
| GUCY1A2 | -1.1695242 | 0.00191286 | guanylate cyclase 1 soluble subunit alpha 2 |
| ABCA6 | -1.1824764 | 0.02699116 | ATP binding cassette subfamily A member 6 |
| GPR75 | -1.1950453 | 0.00185794 | G protein-coupled receptor 75 |
| SIDT1 | -1.2019987 | 0.00048262 | SID1 transmembrane family member 1 |
| SLC26A2 | -1.211629 | 0.00626401 | solute carrier family 26 member 2 |
| GYS2 | -1.2245938 | 0.02065278 | glycogen synthase 2 |
| ANKRD36B | -1.2261883 | 0.01585551 | ankyrin repeat domain 36B |
| B3GAT2 | -1.2367011 | 0.0010803 | beta-1,3-glucuronyltransferase 2 |
| SBSPON | -1.2560329 | 0.00390115 | somatomedin B and thrombospondin type 1 domain containing |
| NEXMIF | -1.2605068 | 0.01099306 | neurite extension and migration factor |
| FSTL4 | -1.2773037 | 0.01458399 | follistatin like 4 |
| SLC2A12 | -1.3018214 | 4.77E-06 | solute carrier family 2 member 12 |
| FETUB | -1.3035143 | 0.01384292 | fetuin B |
| PSMD6-AS2 | -1.3069483 | 0.00692812 | PSMD6 antisense RNA 2 |
| SLFN12L | -1.3170697 | 0.00109241 | schlafen family member 12 like |
| EDN1 | -1.3188519 | 0.00956603 | endothelin 1 |
| ANKRD36C | -1.321415 | 0.00437199 | ankyrin repeat domain 36C |
| LOC101929162 | -1.3223826 | 0.01851508 | uncharacterized LOC101929162 |
| PDGFD | -1.3338043 | 0.0002492 | platelet derived growth factor D |
| CRYM | -1.334357 | 0.03223326 | crystallin mu |
| CYP4F35P | -1.3522682 | 0.00226098 | cytochrome P450 family 4 subfamily F member 35, pseudogene |
| DCN | -1.3663366 | 0.00457119 | decorin |
| PDE1A | -1.3864442 | 0.00185794 | phosphodiesterase 1A |
| PART1 | -1.4097501 | 0.00683807 | prostate androgen-regulated transcript 1 |
| MASP2 | -1.4320308 | 0.00027882 | MBL associated serine protease 2 |
| LRRC7 | -1.4379923 | 0.0163339 | leucine rich repeat containing 7 |
| LNC-LBCS | -1.449672 | 0.00345178 | lncRNA bladder and prostate cancer suppressor, hnRNPK interacting |
| LRRC14 | -1.4915157 | 2.59E-05 | leucine rich repeat containing 14 |
| TPPP3 | -1.4989057 | 0.01426561 | tubulin polymerization promoting protein family member 3 |
| SLC10A5 | -1.5047708 | 0.00879956 | solute carrier family 10 member 5 |
| ALDH1L2 | -1.5603946 | 0.0233677 | aldehyde dehydrogenase 1 family member L2 |
| ALCAM | -1.5958851 | 0.00036811 | activated leukocyte cell adhesion molecule |
| COLEC12 | -1.8365601 | 0.00045659 | collectin subfamily member 12 |
| RNASE7 | -1.9253007 | 0.00668851 | ribonuclease A family member 7 |
| RECQL4 | -1.9447075 | 5.65E-05 | RecQ like helicase 4 |
| FMO9P | -1.9508783 | 6.88E-05 | flavin containing dimethylaniline monoxygenase 9, pseudogene |
| ENPP2 | -1.9616033 | 5.22E-07 | ectonucleotide pyrophosphatase/phosphodiesterase 2 |
| DPYSL3 | -1.9823717 | 0.00076953 | dihydropyrimidinase like 3 |
| FGF14 | -2.0533255 | 0.00049191 | fibroblast growth factor 14 |
| IRAG2 | -2.0623513 | 0.00016617 | inositol 1,4,5-triphosphate receptor associated 2 |
| OXGR1 | -2.110307 | 4.87E-06 | oxoglutarate receptor 1 |
| PAX1 | -2.1369052 | 0.02322487 | paired box 1 |
| MAMDC2 | -2.1512651 | 4.47E-09 | MAM domain containing 2 |
| AQP5 | -2.2038461 | 0.00959934 | aquaporin 5 |
| SLC8A1-AS1 | -2.3295128 | 0.01499802 | SLC8A1 antisense RNA 1 |
| B3GALT5 | -2.5639801 | 0.0053582 | beta-1,3-galactosyltransferase 5 |
| SLITRK5 | -3.367745 | 2.78E-10 | SLIT and NTRK like family member 5 |
